# Load-Carrying Capacity of Hybrid Concrete-Filled FRP Piles

**DOI:** 10.3390/ma19183915

**Published:** 2026-09-15

**Authors:** Sun-Hee Kim

**Affiliations:** Department of Architectural Engineering, Gachon University, Sujeong-gu, Seongnam-si 13120, Republic of Korea; shkim6145@gachon.ac.kr; Tel.: +82-31-750-4718

**Keywords:** compression, bending, load-carrying capacity, hybrid concrete-filled FRP pile, P–M interaction

## Abstract

Piles are structural members subjected to combined compression and bending and are typically constructed in soil or underwater environments. As a result, they are exposed to moisture, chloride attack, and various chemical agents, which may lead to cross-sectional deterioration. These environmental effects can reduce structural durability and should be appropriately considered during the design stage. In practice, compression members are rarely subjected to pure axial loads because eccentricity and lateral loads generally induce combined compression and bending actions. Therefore, the interaction between compression and bending should be carefully considered in structural design. This study experimentally and analytically evaluated the load-carrying capacity of hybrid concrete-filled FRP piles subjected to combined compression and bending. Based on the findings, a P–M interaction diagram was developed to predict the strength and behavior of hybrid concrete-filled FRP piles.

## 1. Introduction

Steel and concrete are the most widely used construction materials in civil engineering due to their economic efficiency and structural durability. Reinforced concrete, in which steel is used to enhance the tensile resistance of concrete, and steel–concrete composite structures have contributed significantly to the advancement in construction materials and have been extensively applied to the construction of long-span and durable structures. However, structural durability gradually deteriorates over time due to exposure to various aggressive environmental conditions, such as carbon dioxide and moisture, which lead to concrete carbonation and steel corrosion. To address these issues, extensive research has been conducted on repair and strengthening techniques to ensure the durability of steel–concrete composite structures, as well as on the development of lightweight advanced materials that can serve as alternatives to steel.

**Figure 1:**
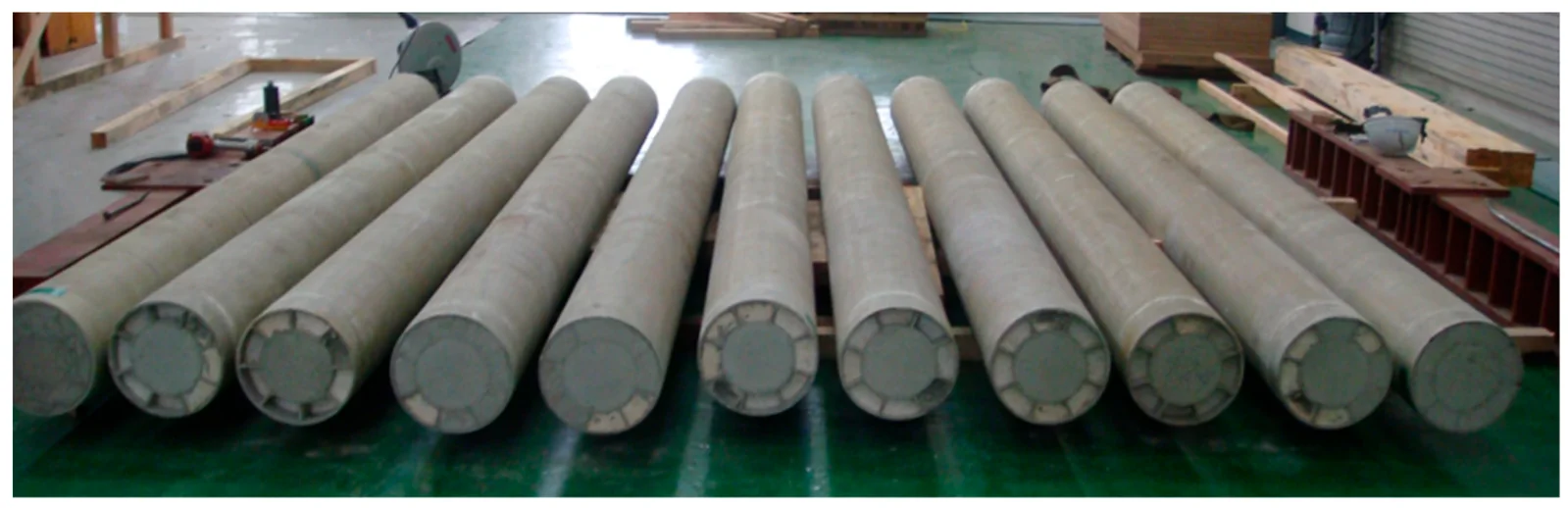
HCFFT pile.

Recently, a new type of pile foundation system using fiber reinforced polymer (FRP) materials has been developed in Korea. The hybrid concrete-filled FRP tube (HCFFT) consists of pultruded fiber reinforced polymer (PFRP) unit modules, the filament winding fiber reinforced polymer (FFRP) arranged in a circular configuration outside the mandrel, and core concrete cast inside the circular tube-shaped hybrid FRP pile [1], as shown in Figure 1.

Moreover, this system can be applied to other structural members subjected to axial compression and bending, such as piers and columns. In the previous research, the characteristics of structural behavior under compression and flexure for the HCFFT and its component materials have been investigated through experimental and analytical studies [1,2,3]. However, the axial compression–flexure interaction behavior of the HCFFT, which represents the typical behavior of piles installed underground, has not been sufficiently evaluated; therefore, a comprehensive investigation is required.

In a study on the axial compression–bending interaction behavior of piles, Zhou et al. [4] conducted lateral load tests on SCC-P piles incorporating steel casing and SCC-F piles with steel casing applied along the entire pile length. Furthermore, a parametric study was conducted using ABAQUS finite element modeling to investigate the effects of steel casing length and thickness. When the steel casing sufficiently enclosed the equivalent plastic hinge region of a conventional reinforced concrete (RC) pile, both the lateral load-carrying capacity and ductility were enhanced. However, when the casing length was insufficient, flexural–shear failure occurred at the lower end of the casing due to contact forces, resulting in reduced deformation capacity and ductility. Xu et al. [5] validated their analytical approach by comparing the behavior of a piled composite foundation (PCF) subjected to lateral loading with results obtained from conventional theoretical solutions and finite element analyses. The results indicated that the PCF exhibited significantly greater pile deflection and bending moments than those observed in conventional pile foundations. Xiao et al. [6] investigated the pile–soil stress ratio in a core-reinforced cement–soil mixing pile composite foundation installed beneath embankments. They proposed a stress-ratio calculation method that incorporates the distribution of shaft friction along different pile segments, thereby providing a basis for representing the actual loading conditions of core-reinforced mixing piles beneath high embankments.

Wang et al. [7] carried out full-scale static load tests on three PSC piles with a total length of 33 m, consisting of PHC piles in the upper section and steel pipe piles in the lower section. Using FBG sensors and strain gauges, they measured strain, axial force, and shaft resistance distributions at each loading stage. The results showed that the geometric discontinuity at the pile joint, arising from the difference in cross-sectional dimensions between the PHC pile and the steel pipe pile, influenced the pile–soil interaction. Consequently, the axial force attenuation behavior and shaft resistance distribution varied within the soil layer containing the joint offset.

Fam and Rizkalla investigated the structural behavior of circular FRP tubes through a series of parametric experiments. The flexural and axial load-carrying behavior of concrete-filled FRP tubes is influenced by the FRP tube thickness, laminate structure, axial stiffness, and concrete confinement effect. These parameters govern the P–M interaction behavior and failure modes [8]. Gong and Noel [9] investigated the feasibility of using CFFTs for wind turbine towers through the finite element analysis. The load-carrying capacity and stiffness of CFFT wind turbine towers are influenced by the longitudinal fiber ratio, concrete filling ratio, reinforcement ratio, prestressing level, and aspect ratio (h/D). Increasing the longitudinal fiber ratio, reinforcement ratio, and prestressing level was found to improve the structural performance. Based on a parametric analysis using experimental results from previous studies, CFFT was confirmed to be an effective alternative to conventional concrete and steel tower systems. Teng et al. [10] experimentally investigated the structural behavior of Hybrid FRP–concrete–steel double-skin tubular columns and confirmed that the GFRP tube enhances structural performance by providing confinement to the concrete and contributing to shear resistance. Al-Zayadi [11] investigated the behavior of FRP hybrid members composed of FRP, concrete, and steel using the finite element analysis. The finite element analysis results showed good agreement with the experimental results. Also, the axial strength ratio increased as the stiffness of concrete, steel, and FRP increased while it decreased with the increase in hollowness ratio.

Most previous studies on composite piles have focused on the behavior of piles composed of concrete and steel pipes, whereas research on composite members consisting of concrete and fiber-reinforced composite materials remains limited [4,5,6,7]. Therefore, based on previous studies [12,13], the present study investigates the load-resisting performance of composite members composed of glass-fiber-reinforced polymer (GFRP) composites and concrete.

In this study, the load–moment relationship was evaluated using axial compression test results and flexural test results of the HCFFT reported in previous studies [12,13].

To assess the accuracy of the flexural analysis, the analytical results were compared with previously reported experimental results. In the analysis, the PFRP cross-section was considered by separately accounting for the inner arc, outer arc, and ribs. Furthermore, the results obtained using the plastic stress distribution method and the strain compatibility method were compared to evaluate their accuracy and applicability.

The flexural stiffness is determined based on the AISC Specification [14] and previous research results. Finally, the axial compression–flexure (P–M) interaction curve for HCFFT members is proposed, and the characteristics of the axial compression–flexure interaction behavior of the HCFFT are investigated based on their compressive and flexural behavior.

## 2. Materials and Methods

### 2.1. Overview

The composition of the HCFFT was developed considering manufacturability, workability, and structural behavior. The HCFFT consists of concrete, FFRP, and PFRP. The PFRP is placed in the core concrete to resist bending moments induced by eccentric loading. This section presents a summary of the experimental results on the mechanical properties of the materials.

### 2.2. Composition and Characteristics

The HCFFT is a composite member reinforced with an outer FFRP layer and an inner PFRP embedded within the concrete element. The externally wrapped FFRP improves the axial load-carrying capacity by providing confinement, while the assembled PFRP modules placed inside enhance the flexural and shear load-carrying capacities.

**Figure 2:**
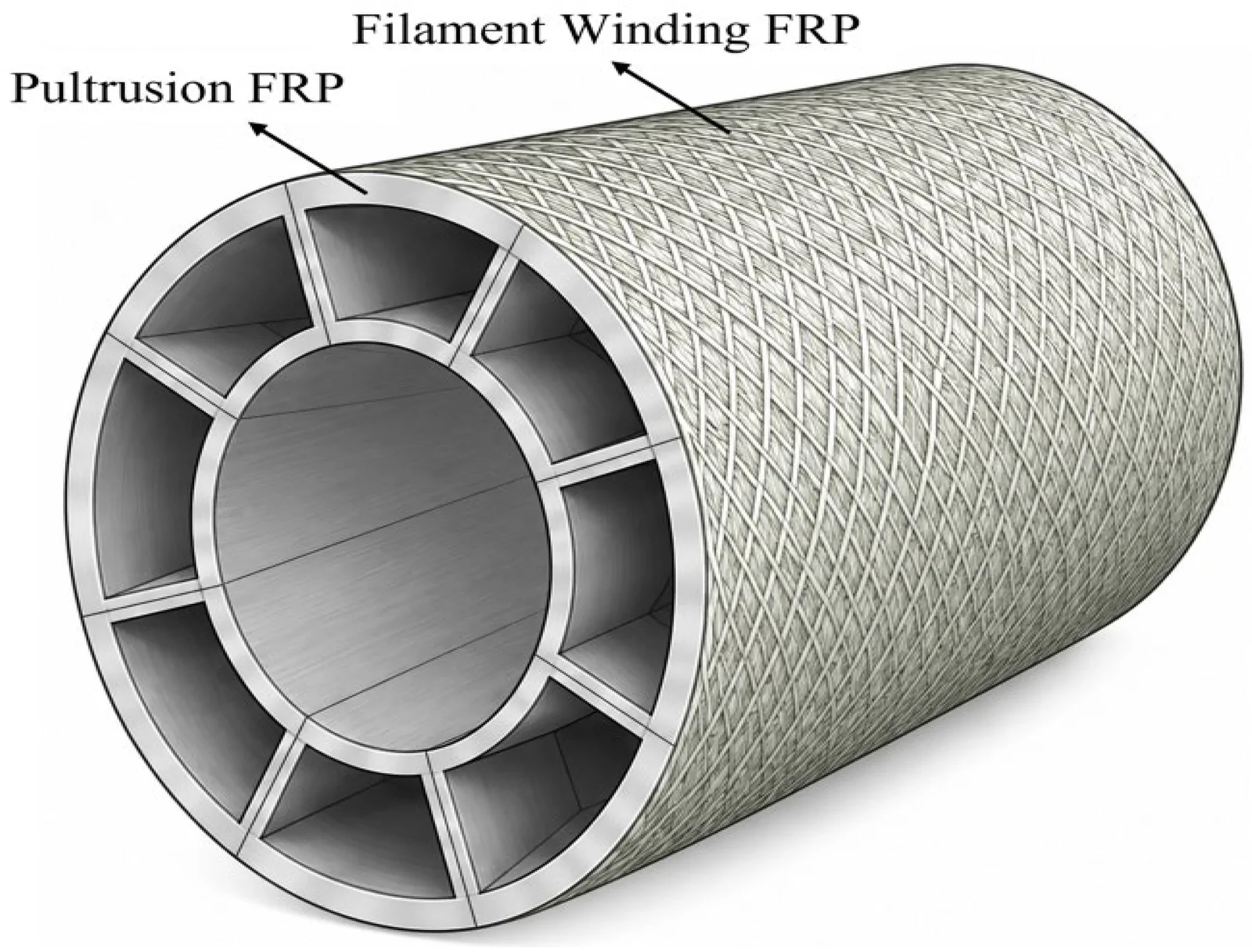
HCFFT shape.

HCFFTs are classified into small- and large-diameter types. In the case of small-diameter HCFFT, the PFRP module system can be manufactured as a single unit covering the entire cross-section. However, for large-diameter HCFFT, PFRP unit segments are assembled to form a circular cross-section. The PFRP modules are then wrapped with an external FFRP layer. In general, due to the characteristics of the pultrusion process, the mechanical properties of PFRP decrease as the cross-sectional size increases. The HCFFT system consists of filament-wound FRP and pultruded FRP, as shown in Figure 2.

### 2.3. Concrete Compressive Strength Test

**Figure 3:**
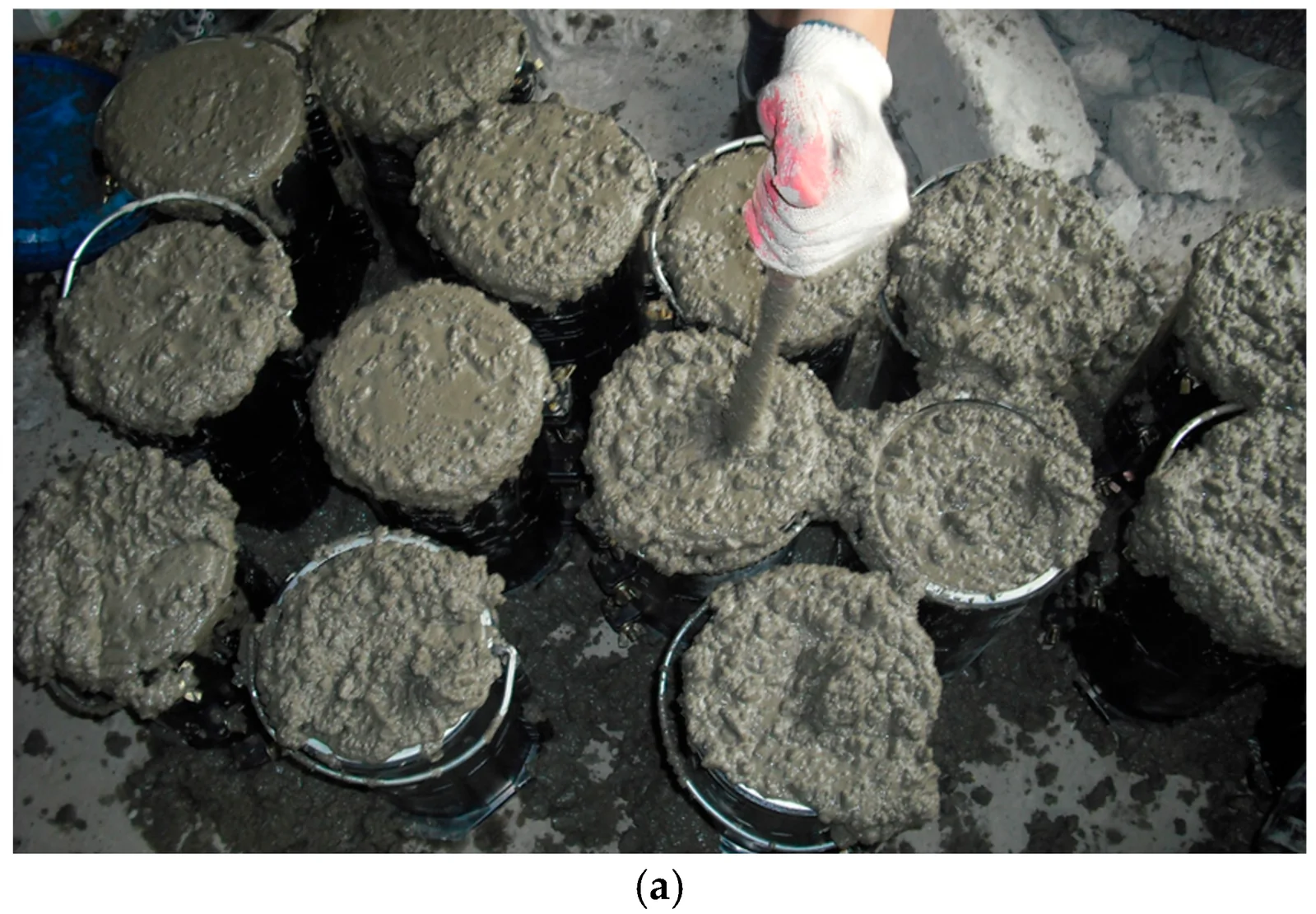

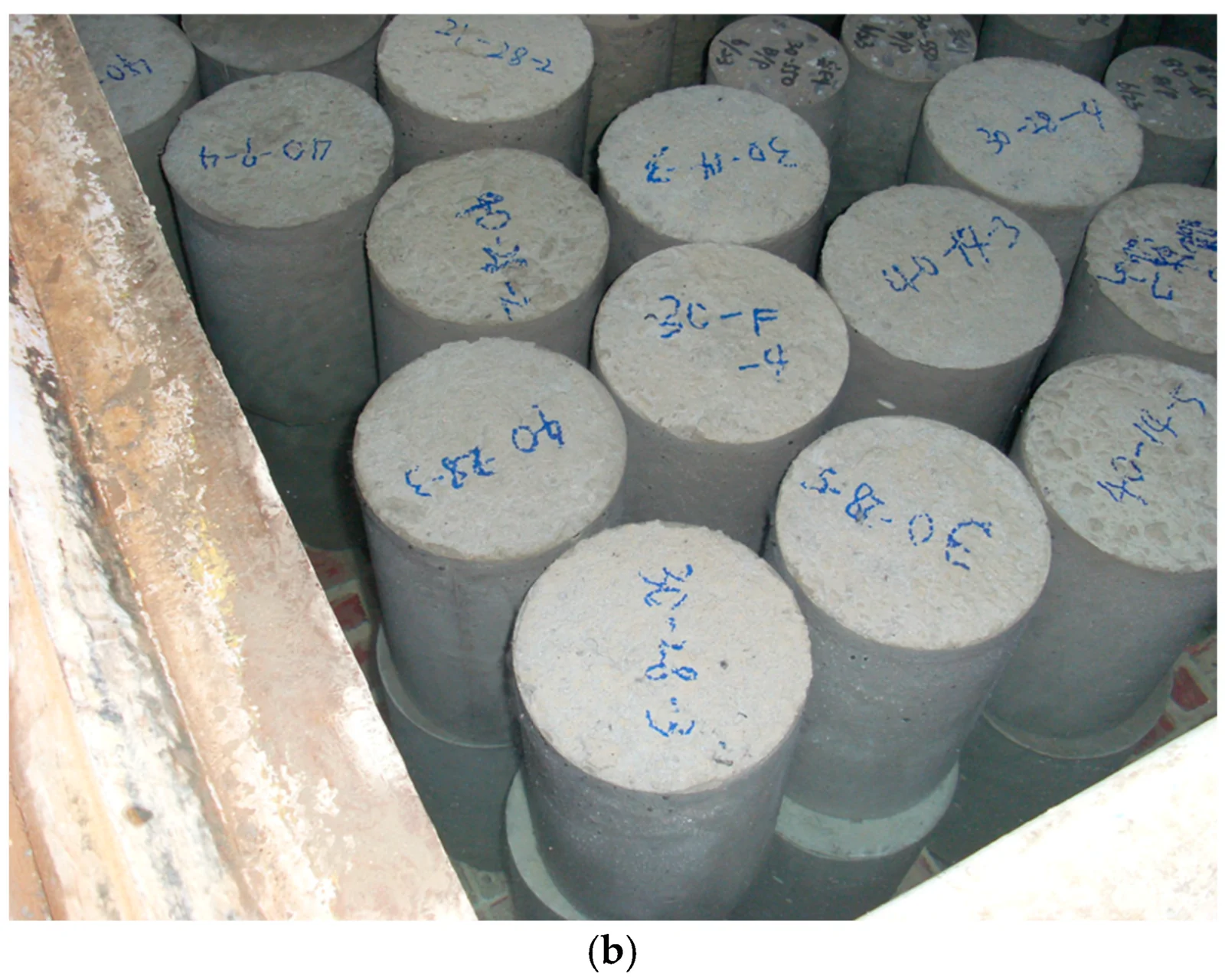
Concrete compression strength test specimens. (**a**) Concrete compaction. (**b**) Water curing.

To evaluate the mechanical properties of the core concrete in the HCFFT, specimens were fabricated and compressive strength tests were conducted. The specimens were produced in a size of ø150 × 300 mm, with 15 specimens for each design compressive strength, and were water-cured. Compressive strength tests were performed at ages of 7, 14, and 28 days after casting. Table 1 presents the types and quantities of the fabricated concrete specimens, and Figure 3 shows the concrete specimens.

The longitudinal compressive displacement of each specimen was measured using a linear variable differential transformer (LVDT) with a measurement range of 100 mm, while the compressive load was applied using a universal testing machine (UTM). The load was applied under displacement-controlled loading at a rate of 1 mm/min.

**Figure 4:**
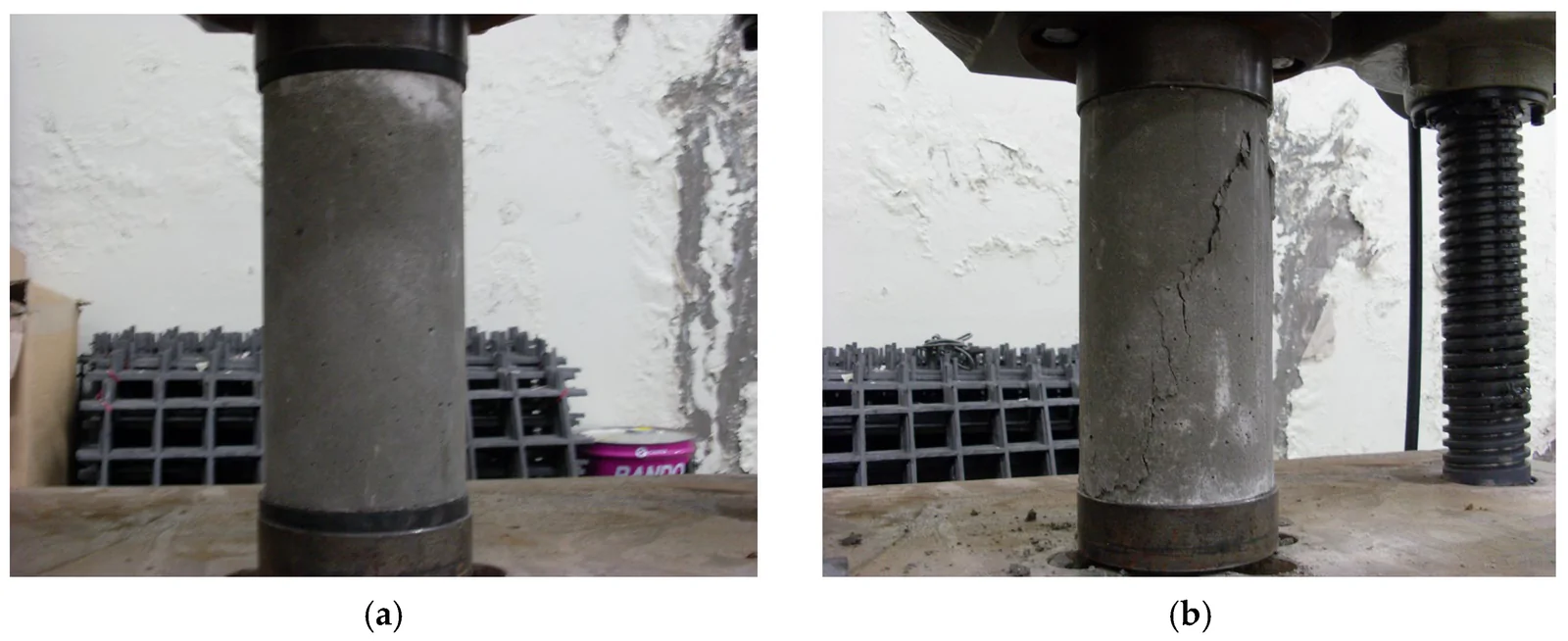
Concrete compression strength test ($f_{ck}=21$ MPa). (**a**) Concrete specimen setup. (**b**) Failure mode.

Figure 4a shows the experimental setup for the compressive strength test, whereas Figure 4b presents the failure mode of the concrete specimens. As shown in Figure 4b, the specimens failed following the initiation and propagation of surface cracks.

The compressive strengths measured after 28 days of curing were comparable to or higher than the specified design compressive strength. The average compressive strengths of the concrete specimens at different curing ages are summarized in Table 2.

## 3. Experiments and Methods

### 3.1. Compressive Strength Test

For the CFFT compressive strength tests, the design compressive strength ($f_{ck}$) of the concrete and the thickness of the FFRP tube ($t_{ffrp}$) were selected as the primary experimental variables. Concrete was cast into FFRP tubes with dimensions of ø150 mm × 300 mm, which were identical to those of the concrete specimens.

The concrete mix proportions are presented in Table 3. The FRP was composed of glass fibers, with which were oriented at an angle of 45°. The pultruded FRP was subsequently wrapped with filament winding FRP to fabricate the composite FRP.

Strain gauges (Tokyo Measuring Instruments Laboratory Co., Ltd., Tokyo, Japan) were attached at the mid-height of each specimen in both the longitudinal and transverse directions. In addition, a displacement transducer (Tokyo Measuring Instruments Laboratory Co., Ltd., Tokyo, Japan) with a measurement range of 100 mm was installed to measure the longitudinal compressive displacement. Compressive loading was applied using a compression testing machine (Sejong, Gyeonggi-do, Republic of Korea) with a capacity of 30,000 kN.

**Figure 5:**
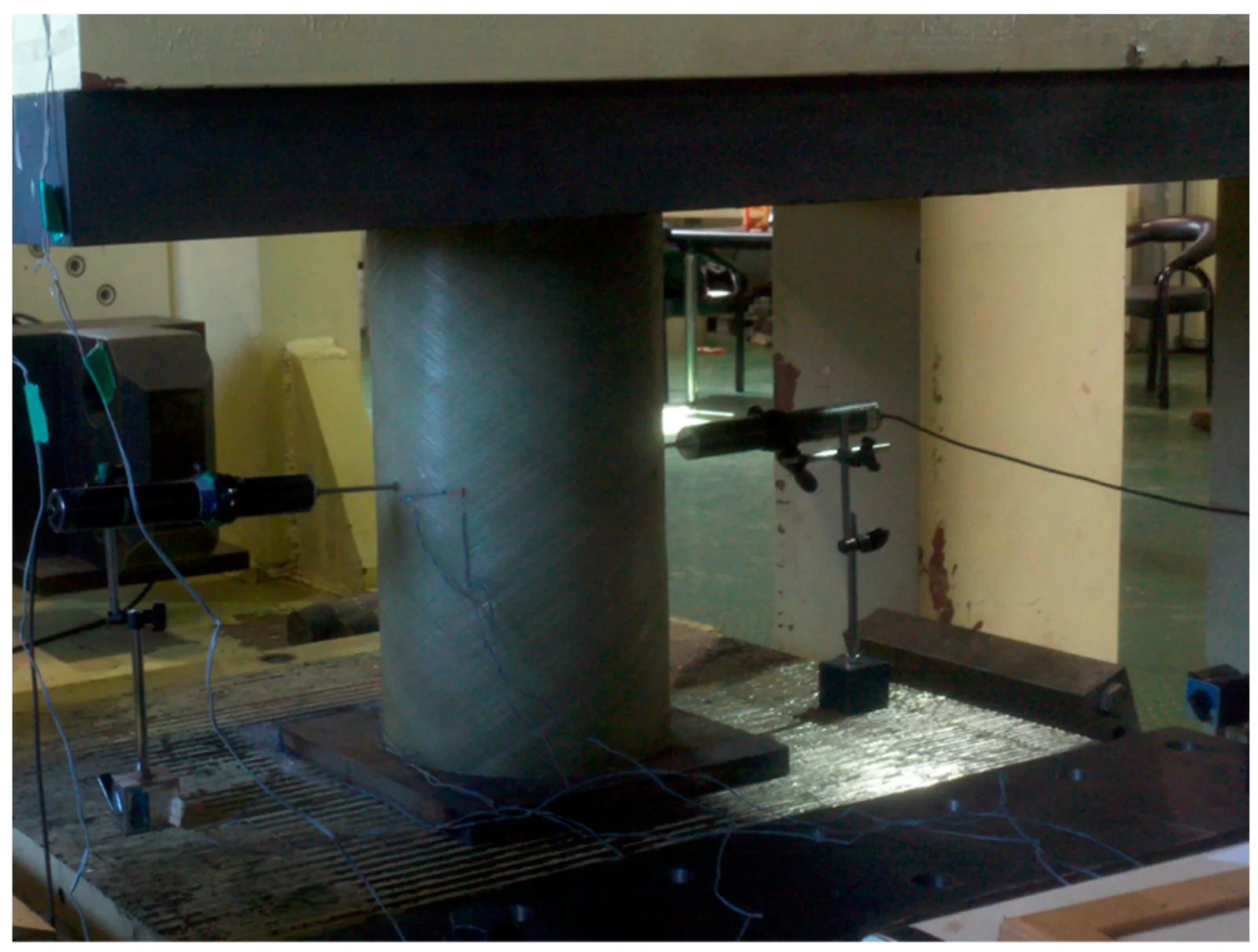
CFFT compressive strength test.

The load was applied under load-controlled conditions at a rate of 300 kN/min, such that the load was transferred only to the concrete core. The experimental setup and loading configuration for the CFFT compressive strength tests are shown in Figure 5.

**Figure 6:**
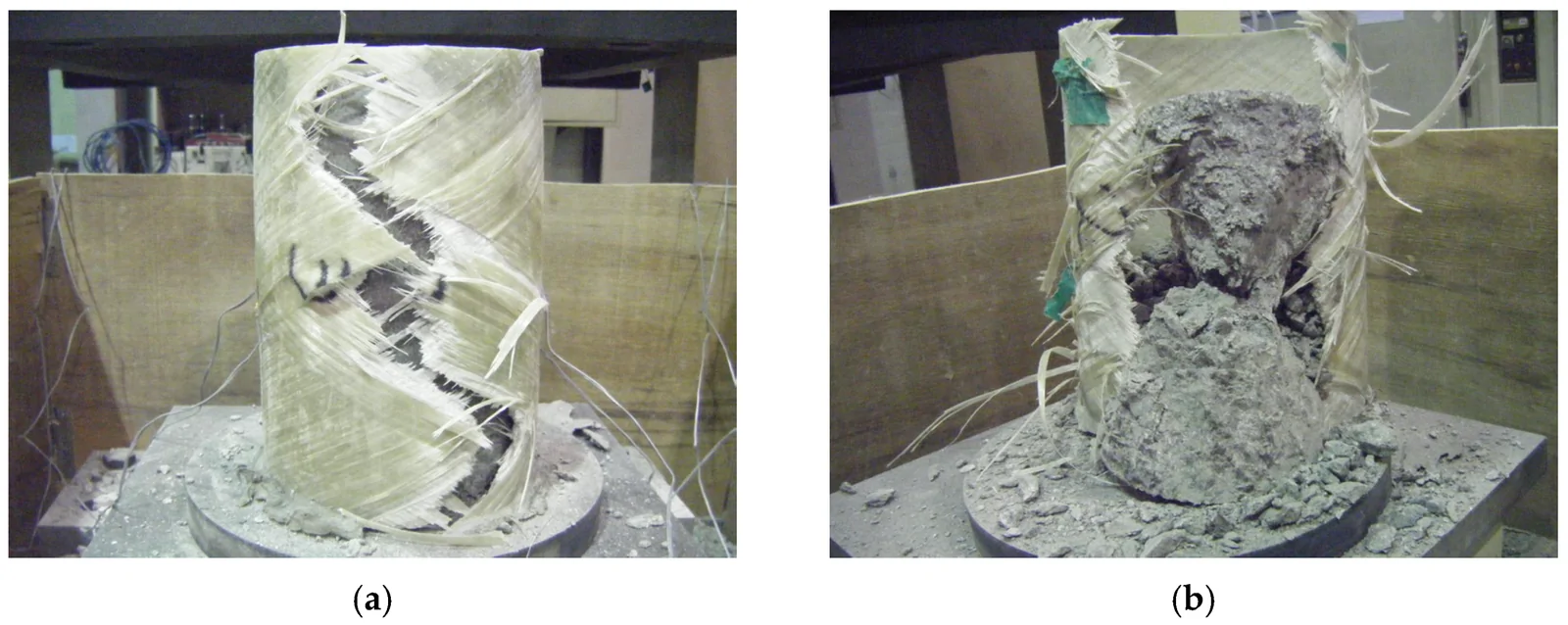
Result of CFFT compression strength test. (**a**) Failure mode (1); (**b**) failure mode (2).

As shown in Figure 6, failure of the specimens occurred through delamination of the FFRP tube or separation of the fiber strands near the mid-height region of the specimens.

The final failure mode of all specimens was brittle failure caused by the circumferential tensile rupture of the FFRP tube. After failure, the internal concrete remained in a symmetrical conical shape at both ends of the specimen, while the remaining portion was crushed and fragmented.

In addition, it was observed that the loading plate, which was installed to transfer the load to the internal concrete, was located below the end of the FFRP tube after testing. This observation suggests that, following the failure of the internal concrete, the FFRP tube continued to resist the applied load, whereas the concrete primarily functioned as an infill material.

**Figure 7:**
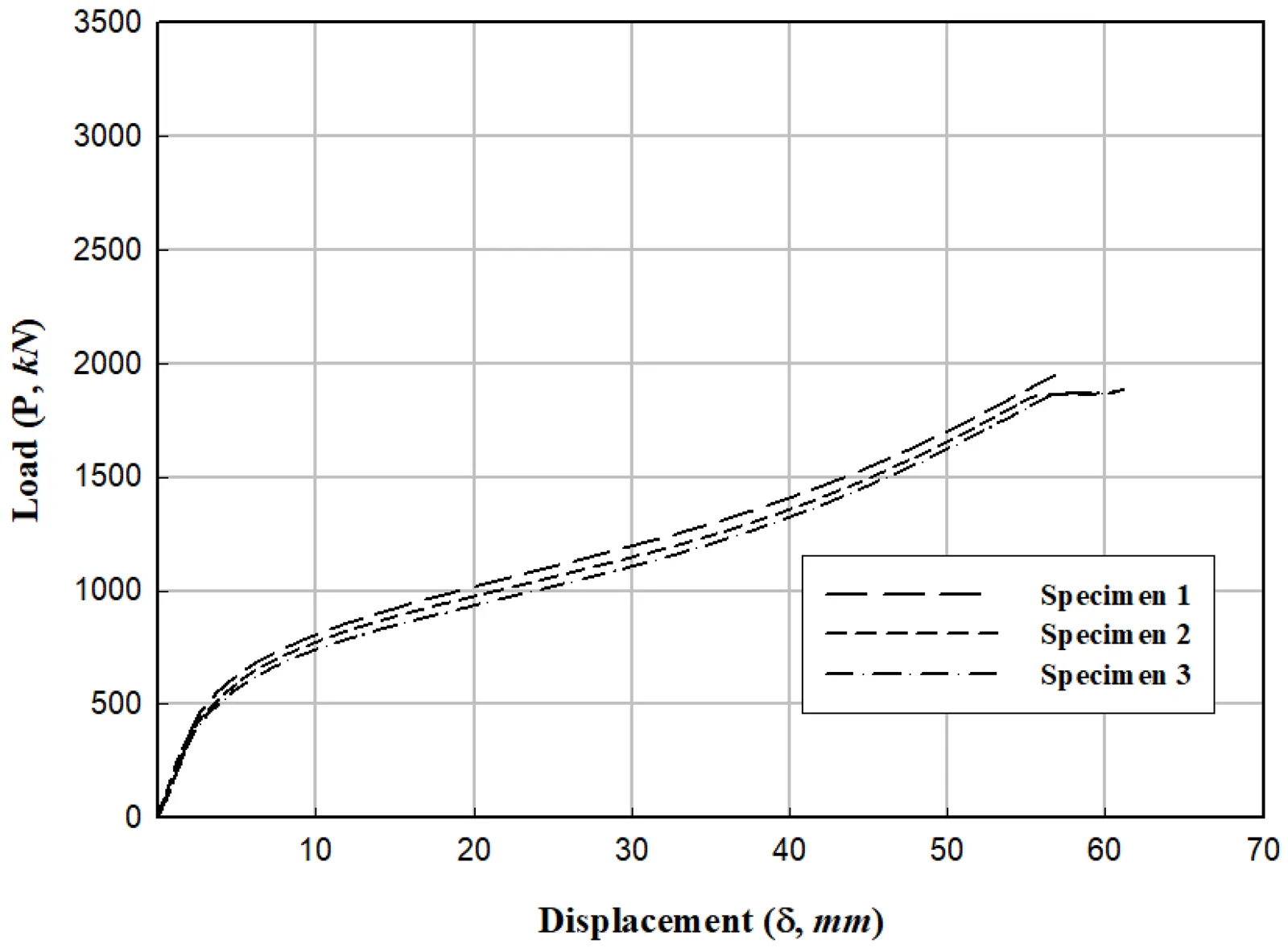
Load–displacement relationship of CFFT (150-21-4.2).

The load–displacement relationships of CFFT are presented in Figure 7. The test results of CFFT are summarized in Table 4.

**Figure 8:**
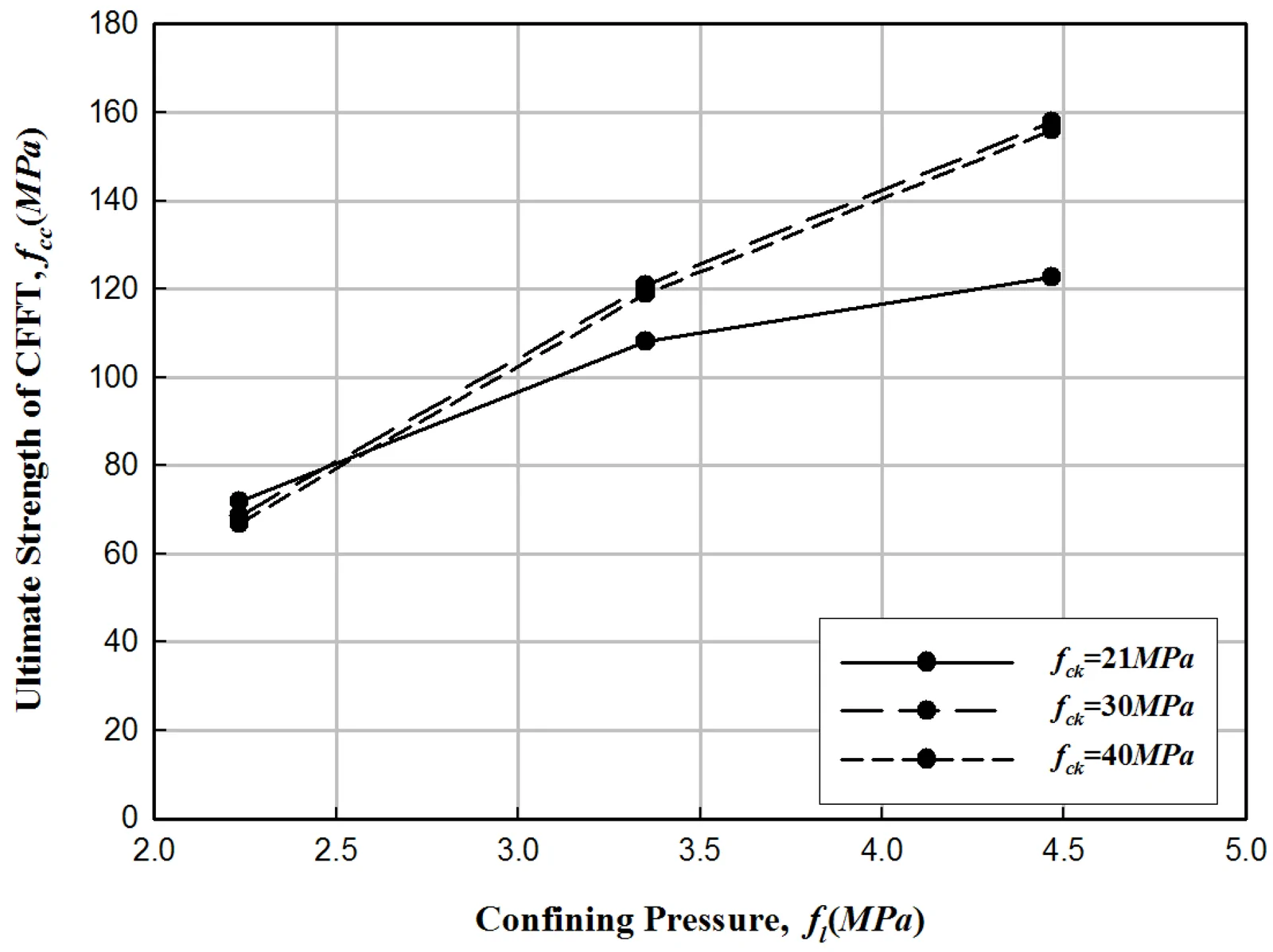
Effect of confinement ratio on the average compressive strength of CFFT specimens.

As shown in Figure 8, the compressive strength increased with increasing confinement pressure associated with the thickness of the FFRP tube. However, when the FFRP thickness was 2.8 mm and the concrete compressive strength exceeded 30 MPa, the strength enhancement effect of the CFFT tended to diminish. This behavior can be attributed to the changes in the mechanical properties of concrete with increasing strength.

**Figure 9:**
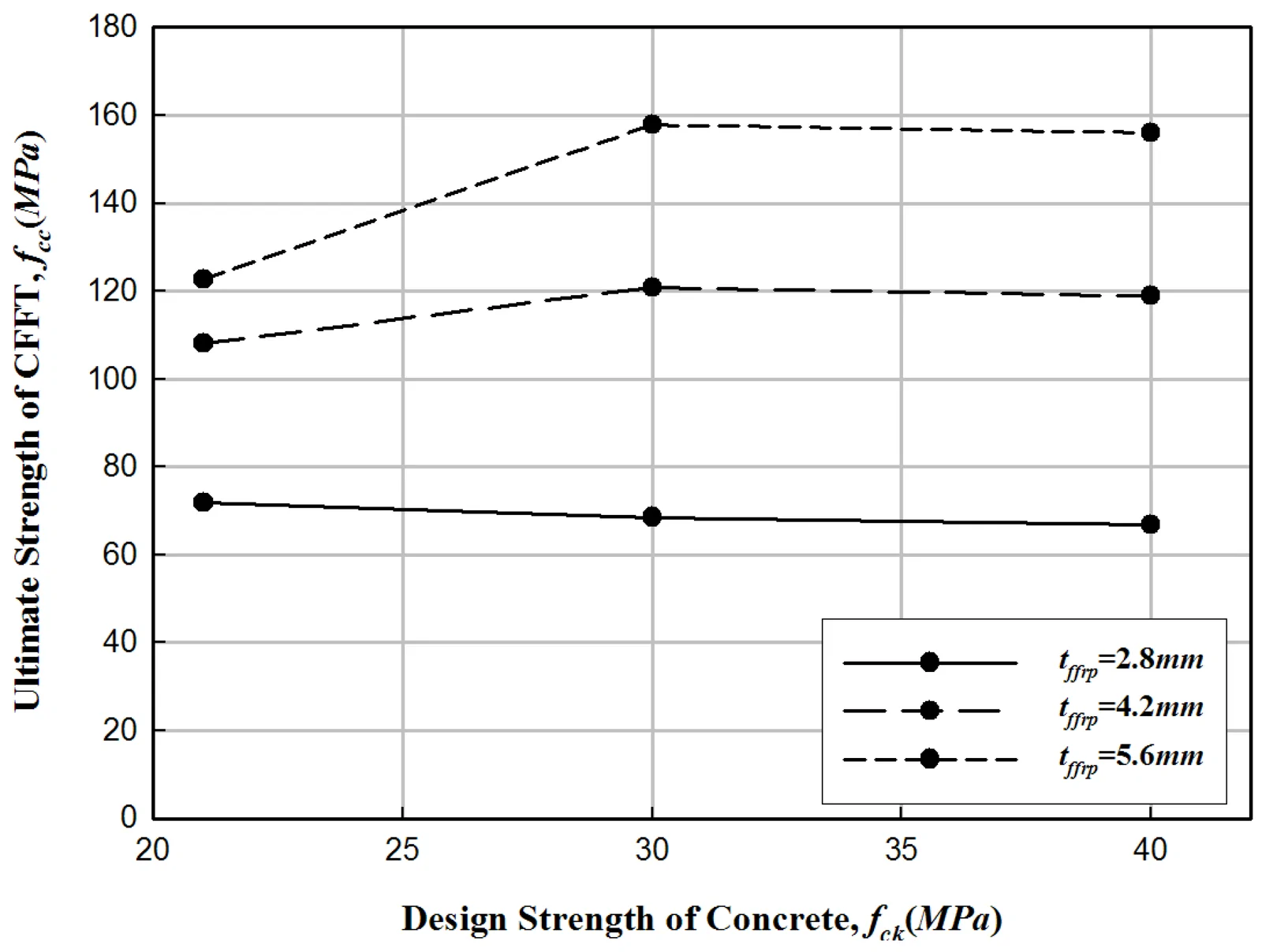
Effect of concrete design compressive strength on the average compressive strength of CFFT specimens.

In addition, Figure 9 shows that the compressive failure strength of CFFT specimens with respect to concrete strength exhibited little variation when the FFRP thickness was 5.6 mm. This behavior is believed to occur because the high confinement pressure provided by the thick FFRP tube governs the failure mechanism, thereby reducing the influence of concrete strength on the ultimate strength of CFFT specimens.

Therefore, it can be concluded that, when the FFRP thickness exceeds a certain threshold, the concrete strength has a negligible effect on the ultimate strength of CFFT.

**Figure 10:**
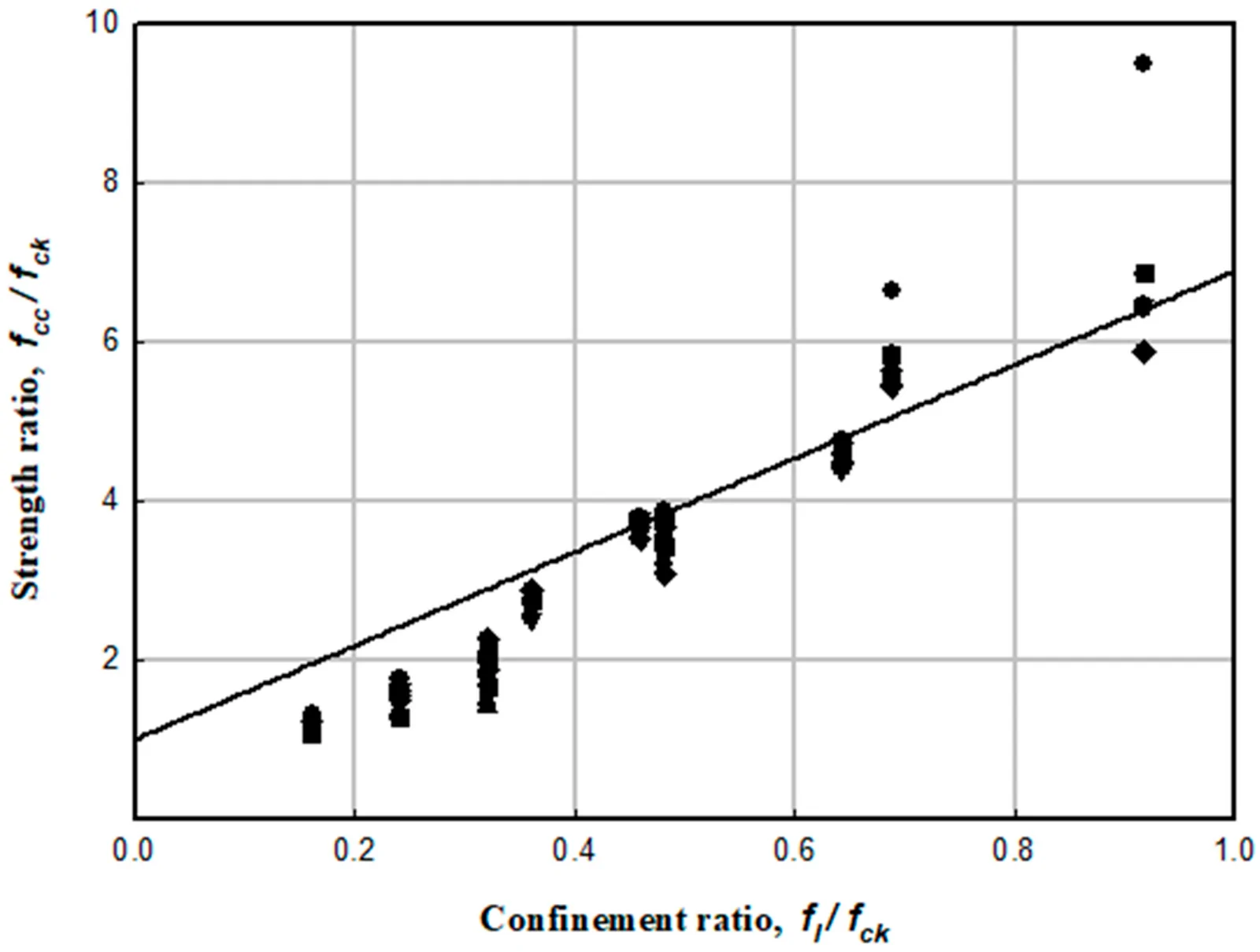
Relationship between the strength ratio and confinement ratio of CFFT specimens.

The relationship between the confinement ratio and strength ratio obtained from the compressive test results of the CFFT specimens is presented in Figure 10. The experimental results showed a linear distribution of data points. A linear regression analysis was performed on the data, and the relationship was expressed as a straight line.

Based on previous studies [12,13], the relationship between the confinement ratio and strength ratio was evaluated using the slope of the regression line, and a compressive strength prediction equation was proposed as shown in Equation (1).

(1) $$\frac{f_{cc}}{f_{ck}}=1.00+5.89\left(\frac{f_{l}}{f_{ck}}\right),$$

where $f_{cc}:$ Represents the compressive strength of the laterally confined concrete column.

$f_{ck}:$ The compressive strength of the column without lateral unconfinement.

$f_{l}:$ The force of lateral confinement.

(2) $$P_{HCFFT}=f_{cc}A_{co}+f_{fr}A_{pfrp},$$

In Equation (2), $f_{fr}$ denotes the compressive stress of the PFRP at the failure of the HCFFT, and $A_{pfrp}$ represents the cross-sectional area of the PFRP.

(3) $$P_{HCFFT}=\frac{P_{HCFFT}^{\prime }}{1.54}=0.65\left(f_{cc}A_{co}+f_{fr}A_{pfrp}\right),$$

Based on Equation (2), a compressive strength prediction equation for the HCFFT was proposed, as shown in Equation (3). The compressive strength values obtained from Equation (3) were compared with the experimental results and are summarized in Table 5. The results showed that the predicted compressive strength values differed from the experimental results by approximately 1.20 to 1.54 times.

Therefore, it is considered that Equation (3) should be applied when using the compressive strength prediction model in design practice.

### 3.2. CFFT Numerical Analysis

In this study, the compressive behavior of CFFT was investigated by analyzing experimental results from compression tests, including the relationship between confinement ratio and strength ratio, as well as the development of compressive strength prediction equations. However, since the experimental variables were limited to a certain range, it is necessary to evaluate whether the findings can be generalized. Therefore, finite element analyses were conducted, and the numerical results were compared with the experimental data.

**Figure 11:**
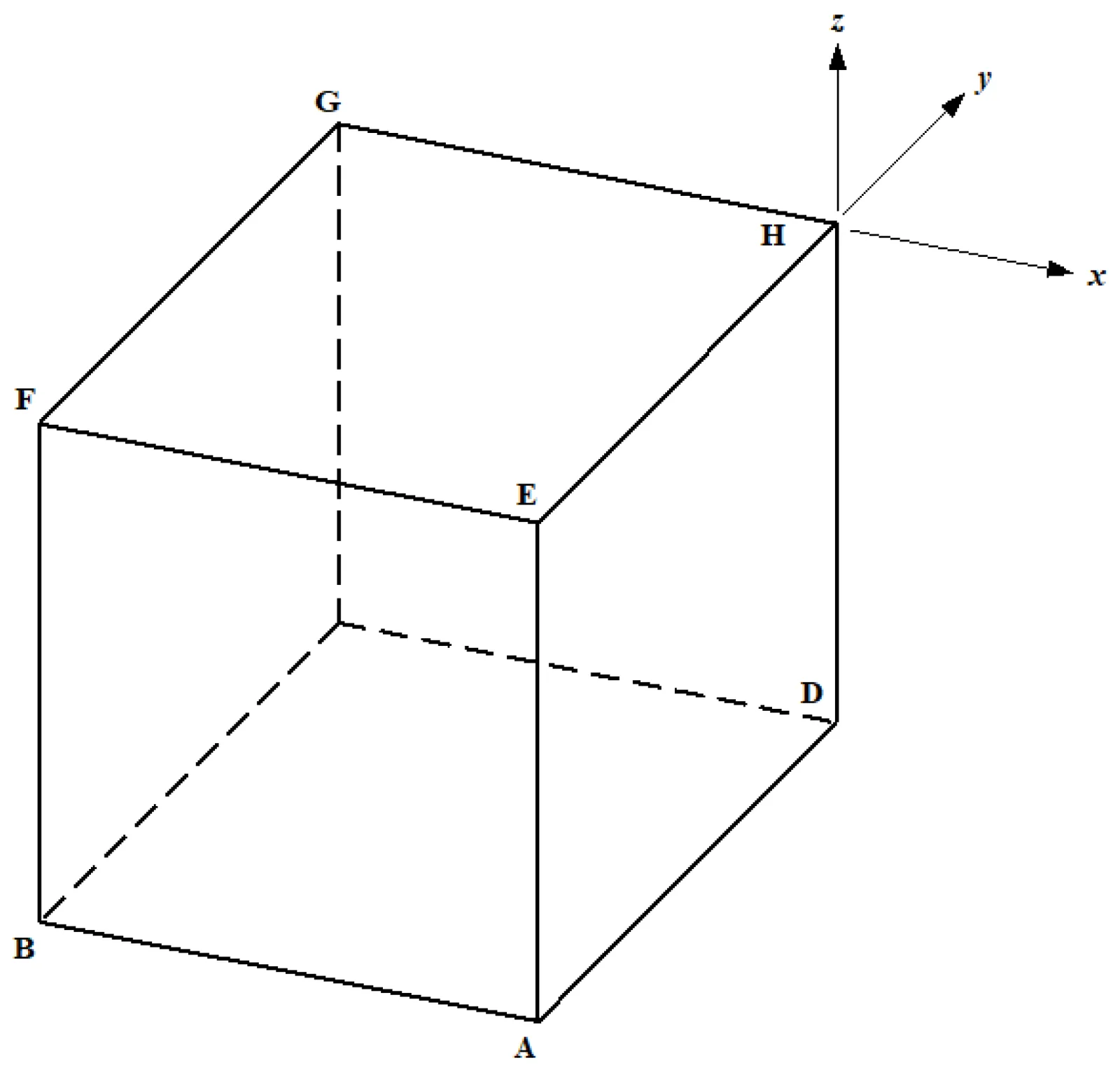
SOLID65 element and degrees of freedom at node H.

The finite element analyses of the CFFT and HCFFT were performed using the general-purpose finite element software ANSYS Ver. 11 [15]. For the HCFFT, the specimen geometry, design compressive strength of concrete, mechanical properties of FRP, and cross-sectional dimensions adopted in previous studies [12,13] were consistently applied. In the finite element analysis, the SOLID65 element provided by ANSYS was employed. The SOLID65 element is used to model reinforced concrete and composite materials such as glass fiber-reinforced composites. As shown in Figure 11, it has eight nodes, and each node has three translational degrees of freedom in the x-, y-, and z-directions [16].

**Figure 12:**
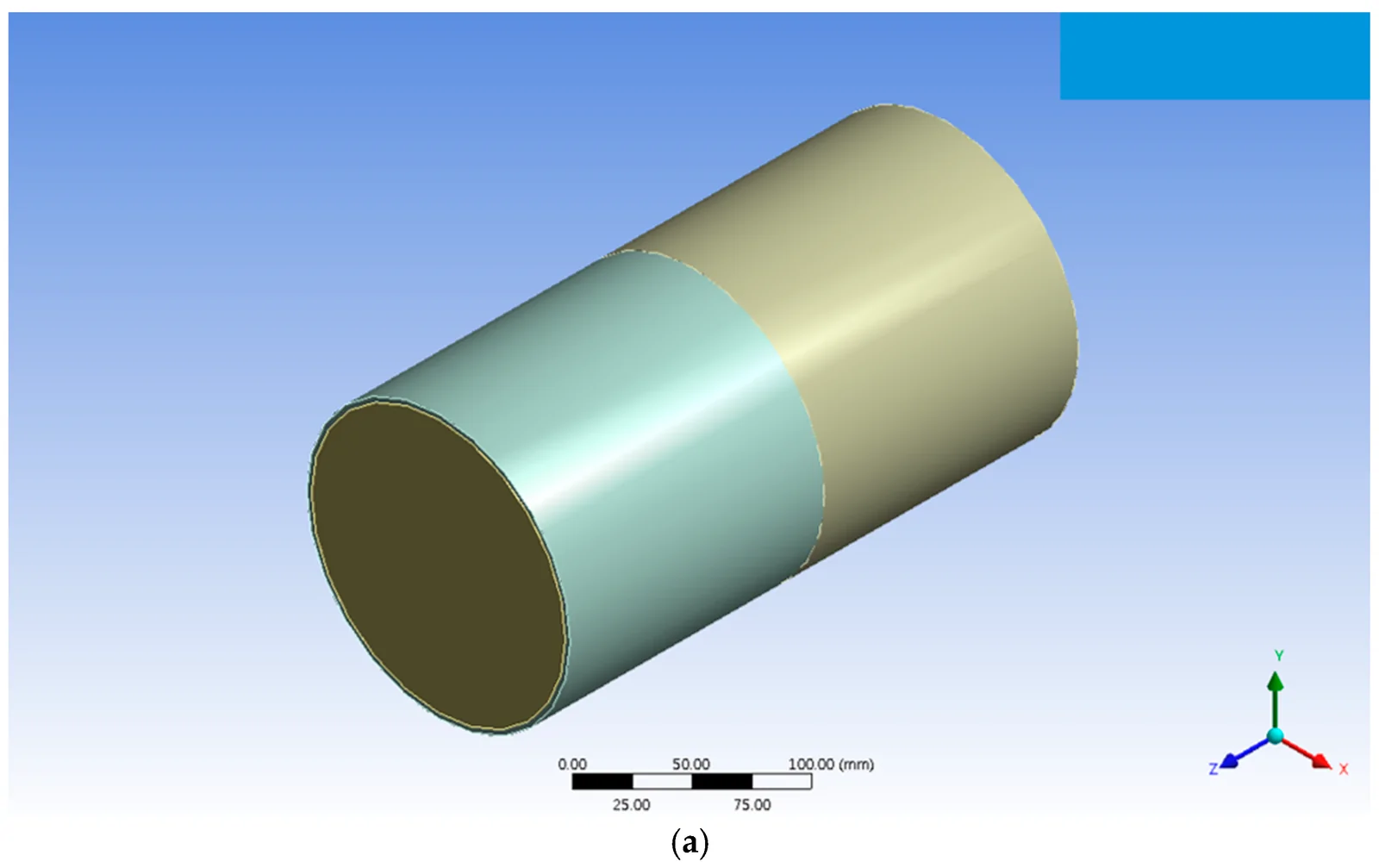

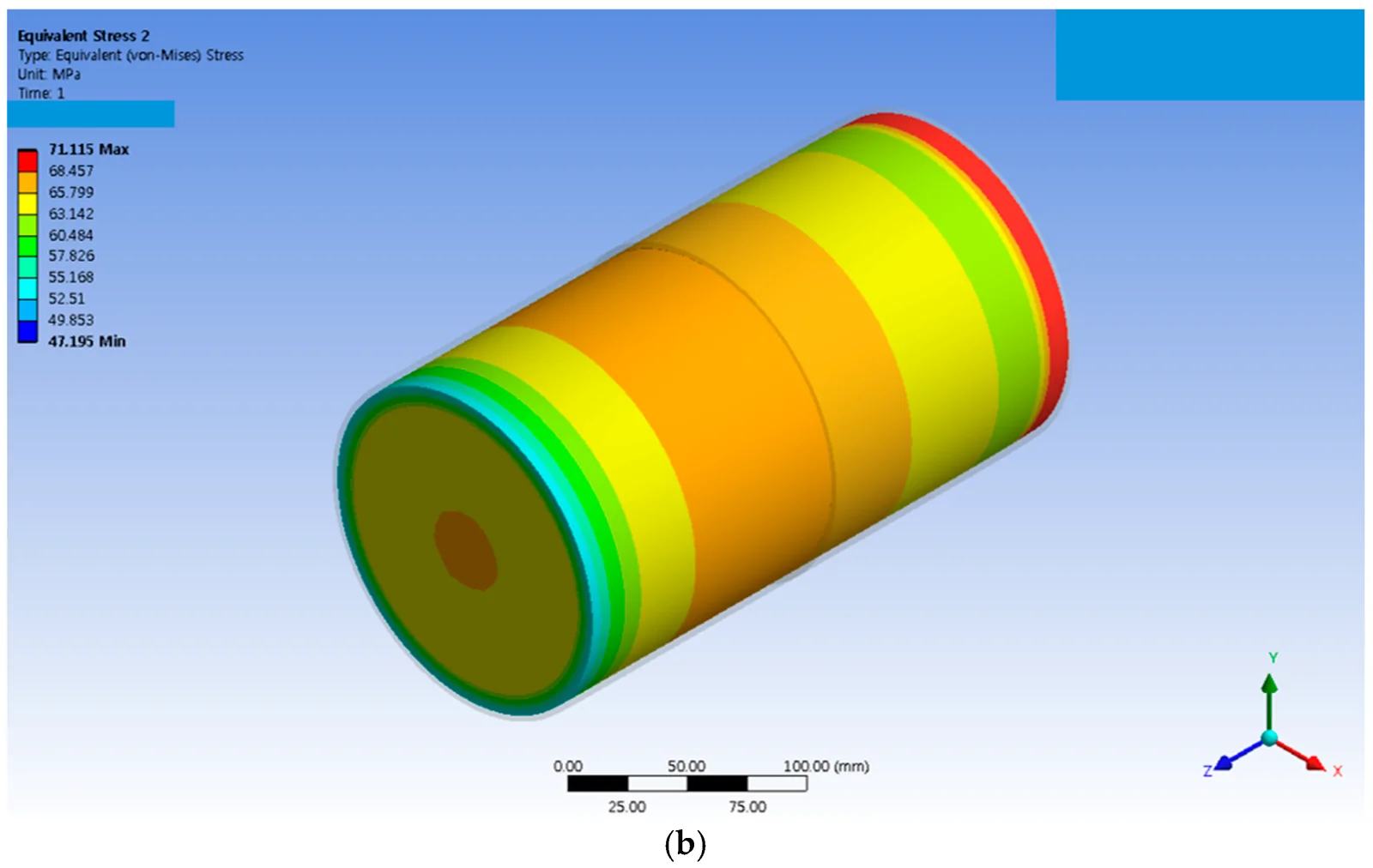

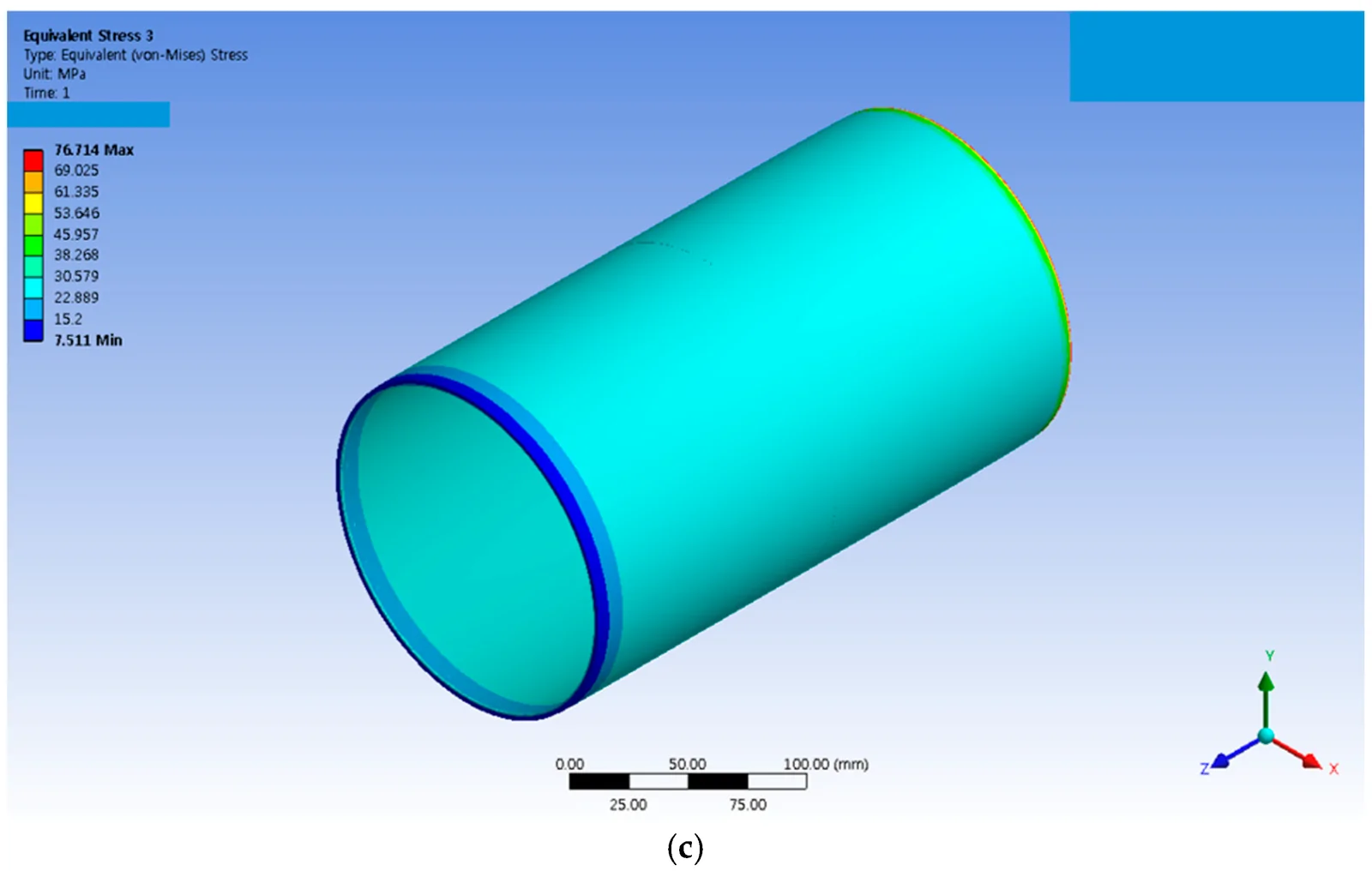
Finite element analysis of CFFT. (**a**) Modeling; (**b**) concrete; and (**c**) FFRP.

The finite element models were developed by directly incorporating the specimen dimensions used in the experiments, the design compressive strength of concrete, and the thickness of the FFRP. The mechanical properties of the materials were obtained from experimental results and applied in the analysis. In addition, the results were evaluated at the central region, which is the farthest point from the boundary conditions and loading points. Figure 12a shows the finite element models of CFFT with diameters of 150 mm and 300 mm, respectively, while Figure 12b,c present the corresponding analysis results for each model.

**Figure 13:**
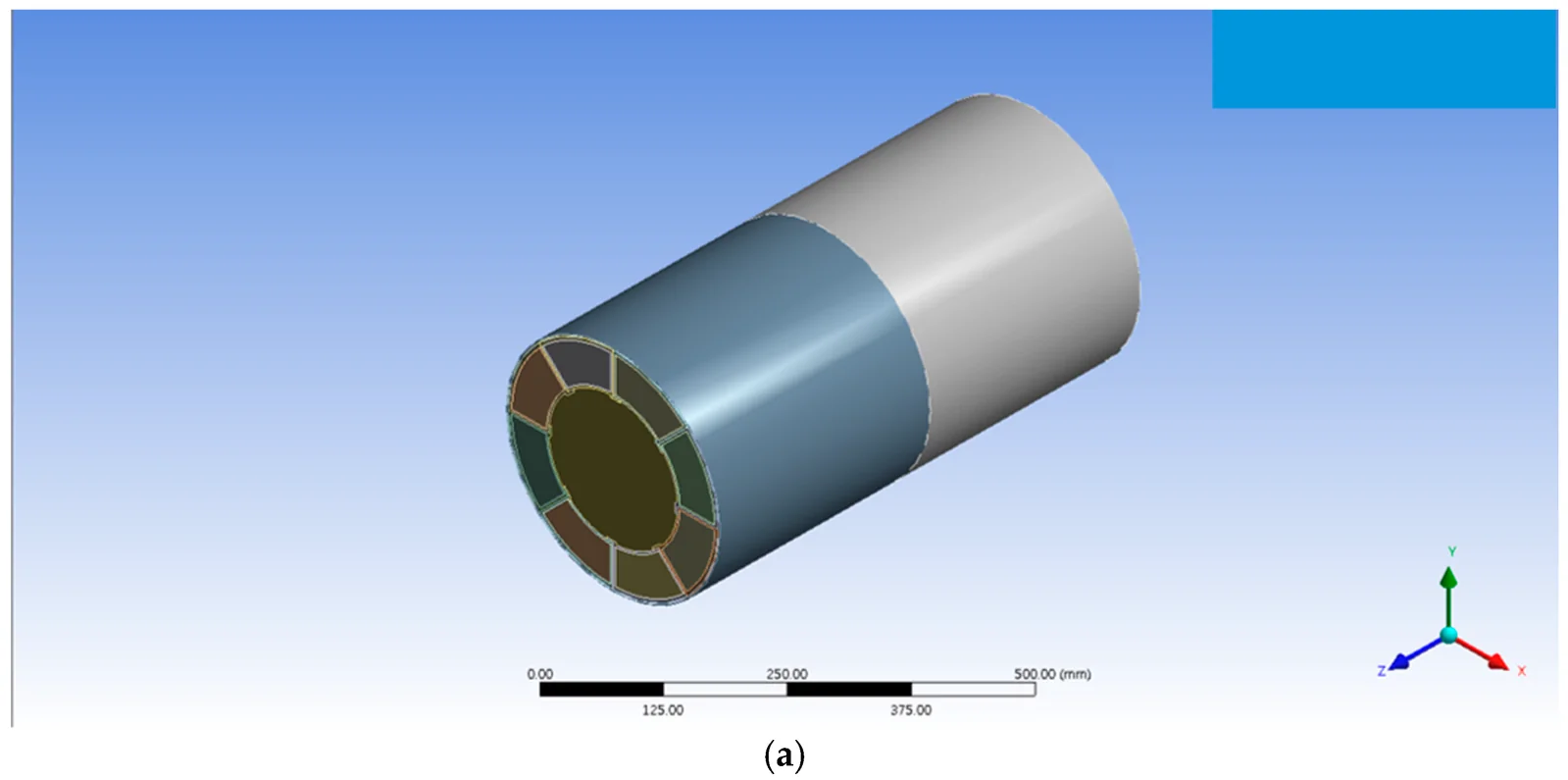

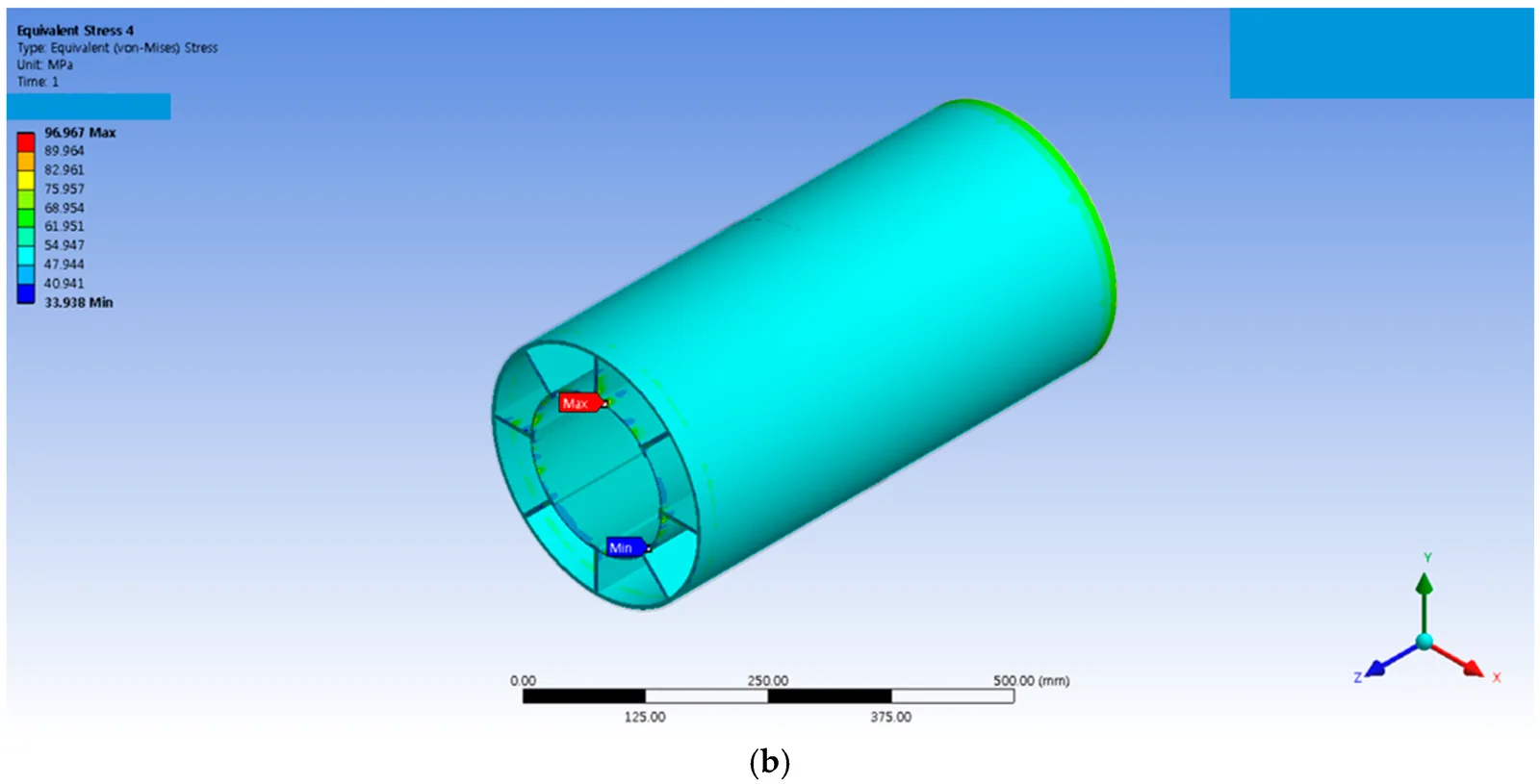

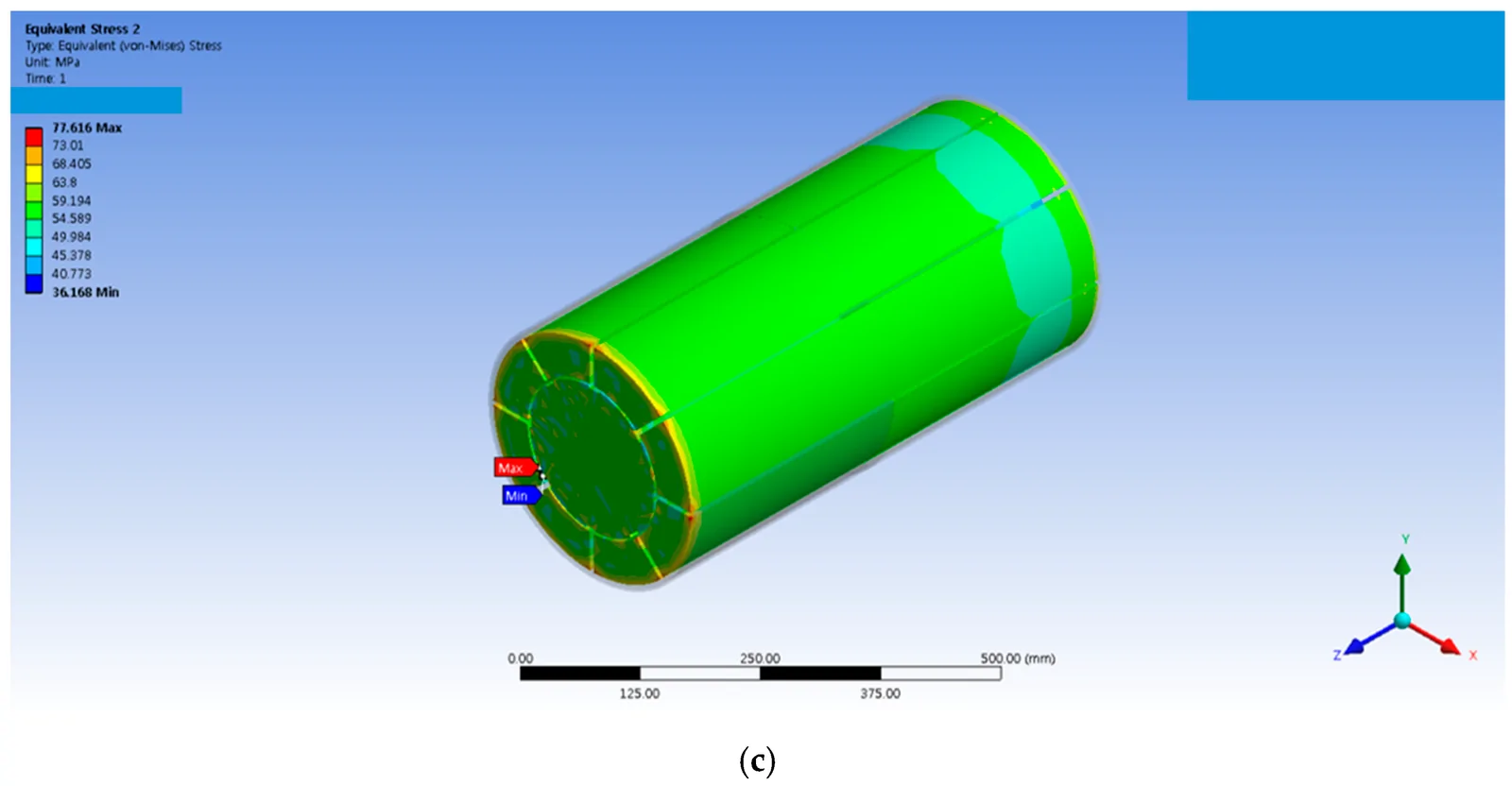

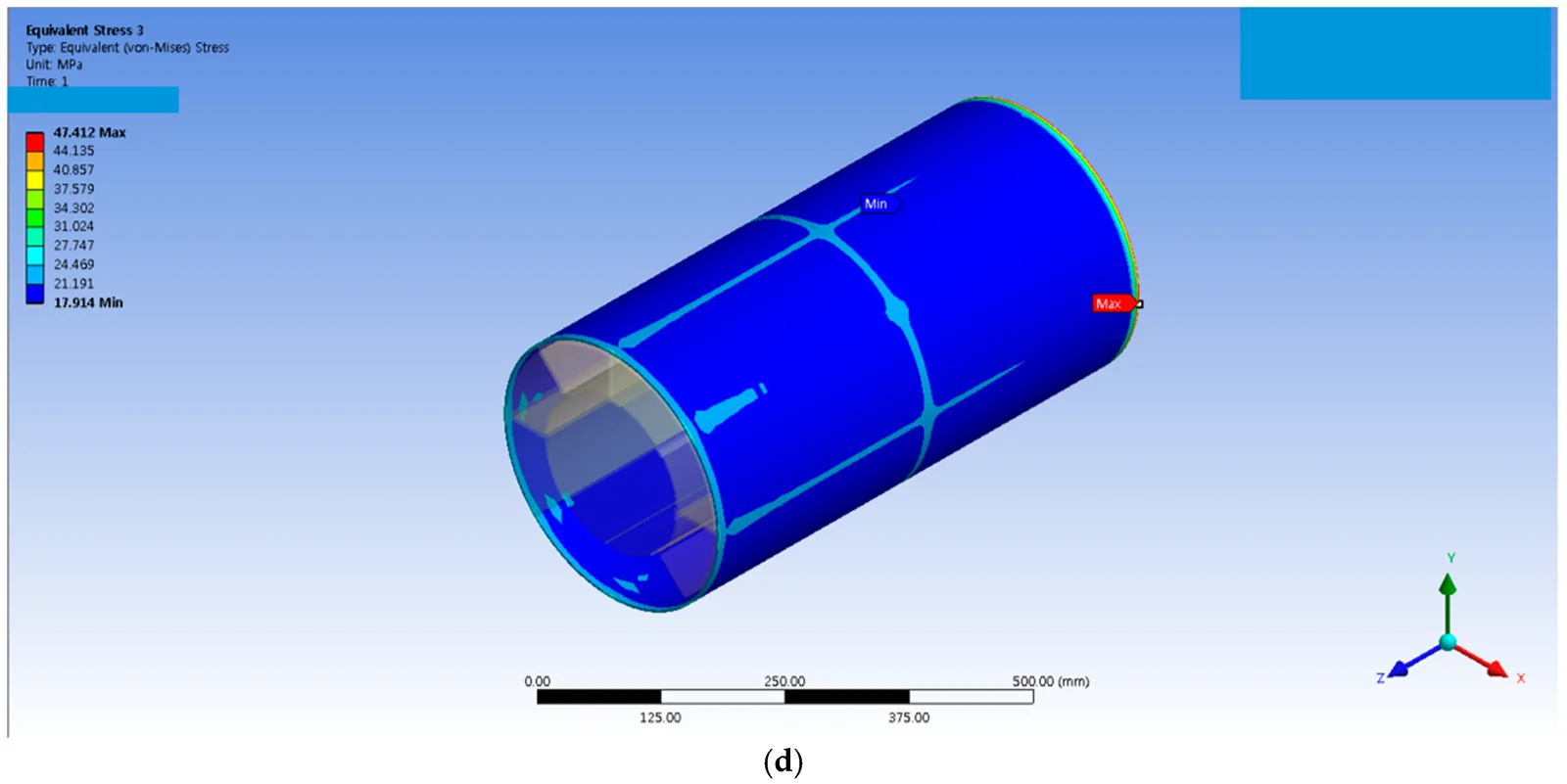
Finite element analysis of the entire HCFFT member. (**a**) HCFFT; (**b**) concrete; (**c**) PFRP; and (**d**) FFRP.

Figure 13 presents the finite element analysis results of the HCFFT. Figure 13a shows the overall model of the HCFFT, Figure 13b presents the failure stress in the concrete. Figure 13 shows the failure stress in the PFRP, and Figure 13d presents the failure stress in the FFRP. Table 6 presents a comparison between the average experimental compressive strength results of HCFFT and the corresponding finite element analysis results according to each variable. The comparison shows that the finite element analysis results exhibit errors ranging from −0.14% to −21.88% relative to the experimental results. Therefore, the numerical model is considered to provide reasonably accurate predictions. In addition, since the compressive strength of CFFT is slightly underestimated, the analysis can be regarded as conservative, thereby ensuring structural safety in design.

### 3.3. Flexural Strength Prediction Equation for HCFFT

**Figure 14:**
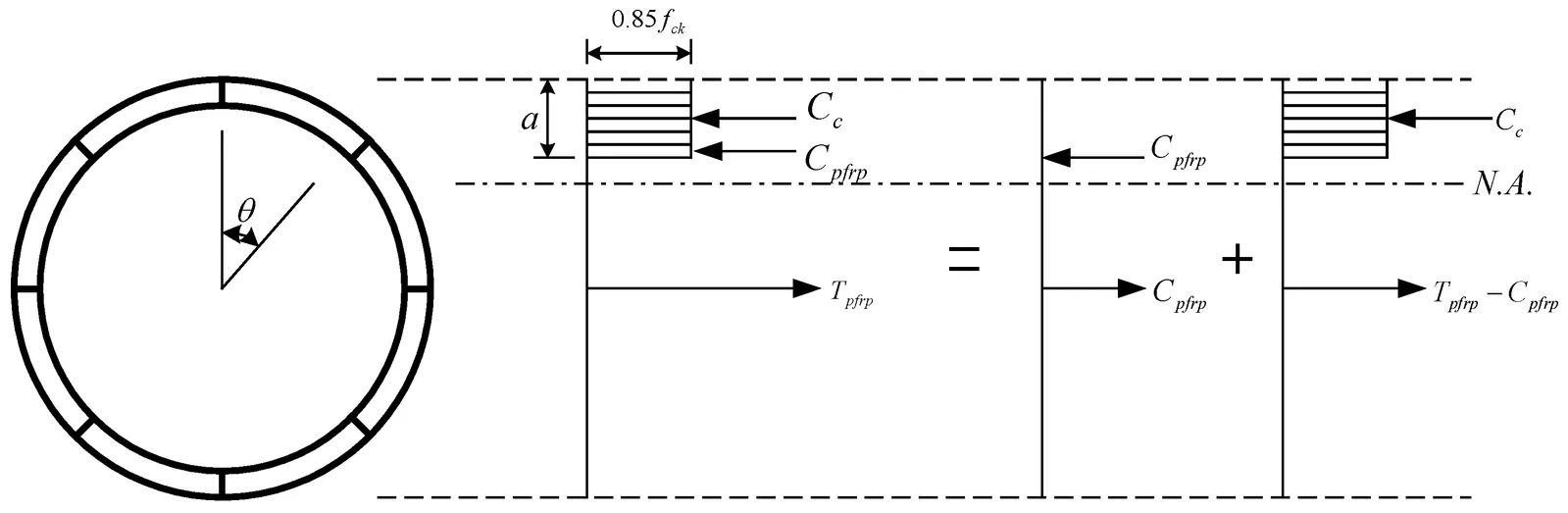
Derivation for HCFFT flexural members from the plastic stress distribution method.

The flexural strength of large-diameter HCFFT can be predicted by applying the equilibrium relationship between tensile and compressive forces in the cross-section induced by the flexural moment [17]. The equilibrium of tensile and compressive forces acting on the cross-section of the HCFFT under flexural loading is illustrated in Figure 14.

As shown in Figure 14, the resisting moment of the cross-section can be divided into the couple moment of PFRP above and below the neutral axis, as well as the couple moment between the compressive concrete and the tensile PFRP. In addition, since the concrete is confined by FFRP, the same confinement coefficient obtained from the HCFFT compressive strength tests was applied. For PFRP, the rib effect of the cross-section cannot be considered due to the variation in the neutral axis; therefore, it was replaced by a ring-shaped section with equivalent cross-sectional properties to account for its compressive strength.

In addition, the flexural strength of the HCFFT was predicted using Equation (4), which was proposed in the previous study [9].

(4) $$M_{HCFFT}=f_{pfrp}r^{3}\left[\left(2\alpha -\pi \right)\left(\frac{\alpha \sin\theta }{\theta \left(\pi -\alpha \right)}+\frac{\sin\theta }{\theta }\right)+\left(\pi -\alpha \right)\left(\frac{\alpha \sin\theta }{\theta \left(\pi -\alpha \right)}+\frac{2\sin^{3}\theta }{3\left(\theta -\sin\theta \cos\theta \right)}\right)\right],$$

In Equation (4), r denotes the radius of the cross-section, and θ represents the angle between the centroid of the cross-section and the radius intersecting the neutral axis. In addition, k is a parameter related to θ, as expressed in Equation (5).

(5) $$\alpha =\theta -\frac{1}{2}\sin\theta ,$$

In this study, the analytical results were compared with the experimental results to verify the accuracy of the proposed flexural analysis equation. In the flexural analysis, the PFRP cross-section was divided into three components: the inner arc, outer arc, and rib.

In addition, to evaluate the contribution of the rib to the flexural load-carrying capacity, the flexural strengths of HCFFTs with and without ribs were compared using Equation (4). The flexural strengths were determined based on the experimental results reported by Kim [13]. Table 7 presents comparison of the flexural strength of HCFFT with and without ribs.

The comparison results indicate that the flexural strength of HCFFTs with ribs differs from that of members without ribs by approximately 3% according to the plastic stress distribution method and by approximately 10% according to the strain compatibility method. Therefore, it can be concluded that the additional flexural resistance provided by the rib has a relatively minor influence on the overall flexural strength of HCFFT.

**Table 7:** Comparison of experimental and analytical flexural stregnths of HCFFT.

FFRP Thickness (*t_f_*, mm)	Previous Research of Average Experimental Results ($M_{n\left(exp\right)},\;kNm$)	Analytical Results					
		Plastic Stress Distribution Method			Strain Compatibility Method		
		With Rib (kNm)	Without Rib (kNm)	Without/With (%)	With Rib (kNm)	Without Rib (kNm)	Without/With (%)
2.8	353.1	309.30	300.27	97.08	219.39	196.53	89.58
4.2	351.6	315.81	306.76	97.13	224.77	201.92	89.83
5.6	397.2	322.46	313.38	97.18	230.24	207.42	90.09

## 4. Compression–Flexure Interaction Behavior of HCFFT Member

For the design of compression members, including piles, the flexural stiffness and the compression–flexure interaction behavior need to be considered. By referring to the AISC specification and previous research [4,5,6,12] the equation for the flexural stiffness is suggested. Moreover, the P–M interaction curve for HCFFT members is suggested and the characteristics of compression–flexure interaction behavior of HCFFT members are investigated considering the characteristics of compression and flexural behaviors of HCFFT members.

### 4.1. Flexural Stiffness of HCFFT Members

In the AISC specification [14], the design compressive strength for the axially loaded CFT member shall be determined for the limit state of flexural buckling expressed as Equations (6) and (7).

(6) $$P_{n}=P_{o}\left\{0.658^{{\left(\frac{P_{o}}{P_{e}}\right)}^{2}}\right\}\;at\;P_{e}\geq 0.44P_{o},$$

(7) $$P_{n}=0.877P_{e}\;\;\;at\;\;P_{e}<0.44P_{o},$$

In Equations (6) and (7), $P_{e}$ is the Euler buckling load expressed as Equation (8).

(8) $$P_{e}=\frac{\pi^{2}{EI}_{eff}}{{\left(KL\right)}^{2}},$$

In Equation (8), ${EI}_{eff}$ is the effective rigidity of the cross-section and K is the effective length factor determined by considering the boundary condition of member ends. In the AISC specification [14], the effective stiffness (or rigidity) is suggested by considering the structural characteristics. The effective stiffness for encased composite columns, such as the CFT member, is expressed as Equation (9).

(9) $${EI}_{eff}=E_{s}I_{s}+E_{s}I_{sr}+C_{3}E_{c}I_{c},$$

In Equation (9), $E_{s}$ is the elastic modulus of steel tube, $I_{s}$ is the moment of inertia of steel tube, $I_{sr}$ is the moment of inertia of reinforcing bar, $C_{3}$ is the reduction factor related to the flexural stiffness, $E_{c}$ is the elastic modulus, and $I_{c}$ is the moment of inertia of concrete, respectively, and $C_{3}$ is suggested as Equation (10).

(10) $$C_{3}=0.6+2\left(\frac{A_{s}}{A_{c}+A_{s}}\right)\leq 0.9,$$

In Equation (10), $A_{c}$ and $A_{s}$ are the area of concrete and steel tube, respectively. In the previous research, the effective stiffness and reduction factor of the HCFFT are suggested as given in Equation (11) and Equation (12), respectively.

(11) $${EI}_{HCFFT}=E_{fT}I_{f}+E_{p}I_{p}+C_{H}E_{c}I_{c},$$

(12) $$C_{H}=0.6+2\left(\frac{A_{f}}{A_{c}+A_{f}}\right),$$

In Equations (11) and (12), ${EI}_{HCFFT}$ is the effective stiffness for the HCFFT, $I_{f}$ is the moment of inertia of FFRP, $I_{p}$ is the moment of inertia of PFRP, and $C_{H}$ is the reduction factor related to flexural stiffness, respectively.

In Equations (11) and (12), the reduction factor is a function related to the confinement effect. Therefore, Equation (12) may not be correct because it is derived simply as the factor for CFT is replaced by the factor for the HCFFT member and, accordingly, additional further studies (including experimental and analytical studies) are necessary.

### 4.2. P–M Interaction Curve of HCFFT Member

As described previously, the HCFFT pile is an unreinforced pile without steel reinforcement; therefore, the equations developed for compression members reinforced with steel bars serving as flexural reinforcement cannot be directly applied. The PFRP used as tensile reinforcement in the HCFFT exhibits an approximately linear-elastic response up to failure without a yielding stage. By incorporating the tensile behavior of PFRP, the P–M interaction diagram of the HCFFT can be obtained by replacing the steel yield strength, $f_{y}$, with the ultimate tensile strength of PFRP, $f_{pfrp}$, in Equations (4)–(12).

In this study, the P–M interaction diagrams were developed based on the results of the compression tests and the flexural strength prediction equation proposed by Kim [13]. However, unlike steel reinforcement, PFRP possesses a complex cross-sectional geometry, making it difficult to accurately determine sectional properties, such as the area and centroid of the PFRP located in the tension and compression zones under eccentric loading. Therefore, the sectional properties of PFRP were incorporated into the analysis through numerical modeling of the HCFFT cross-sections fabricated for the experiment.

**Figure 15:**
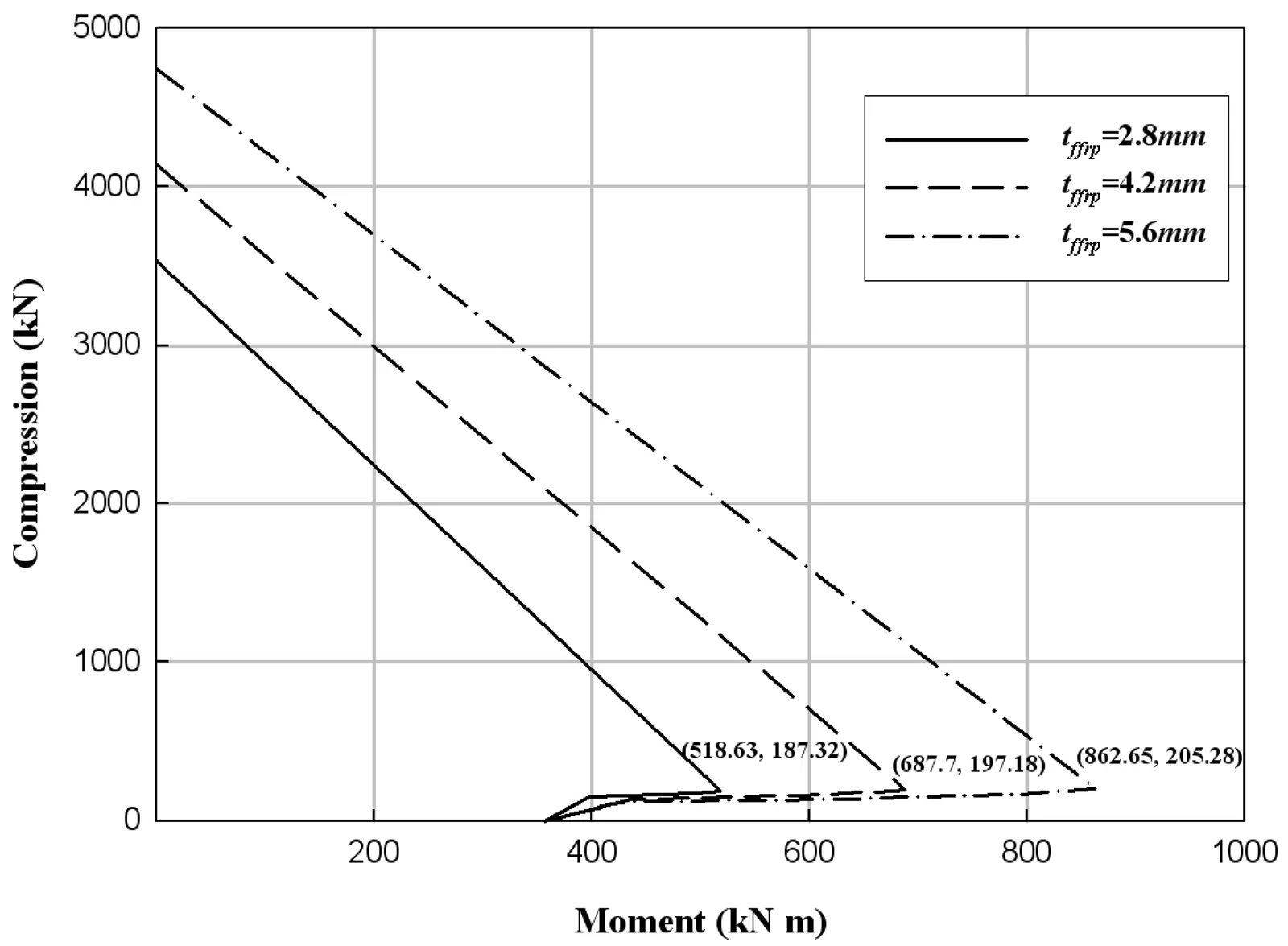
P–M interaction diagram for the 300 mm diameter HCFFT specimen.

**Figure 16:**
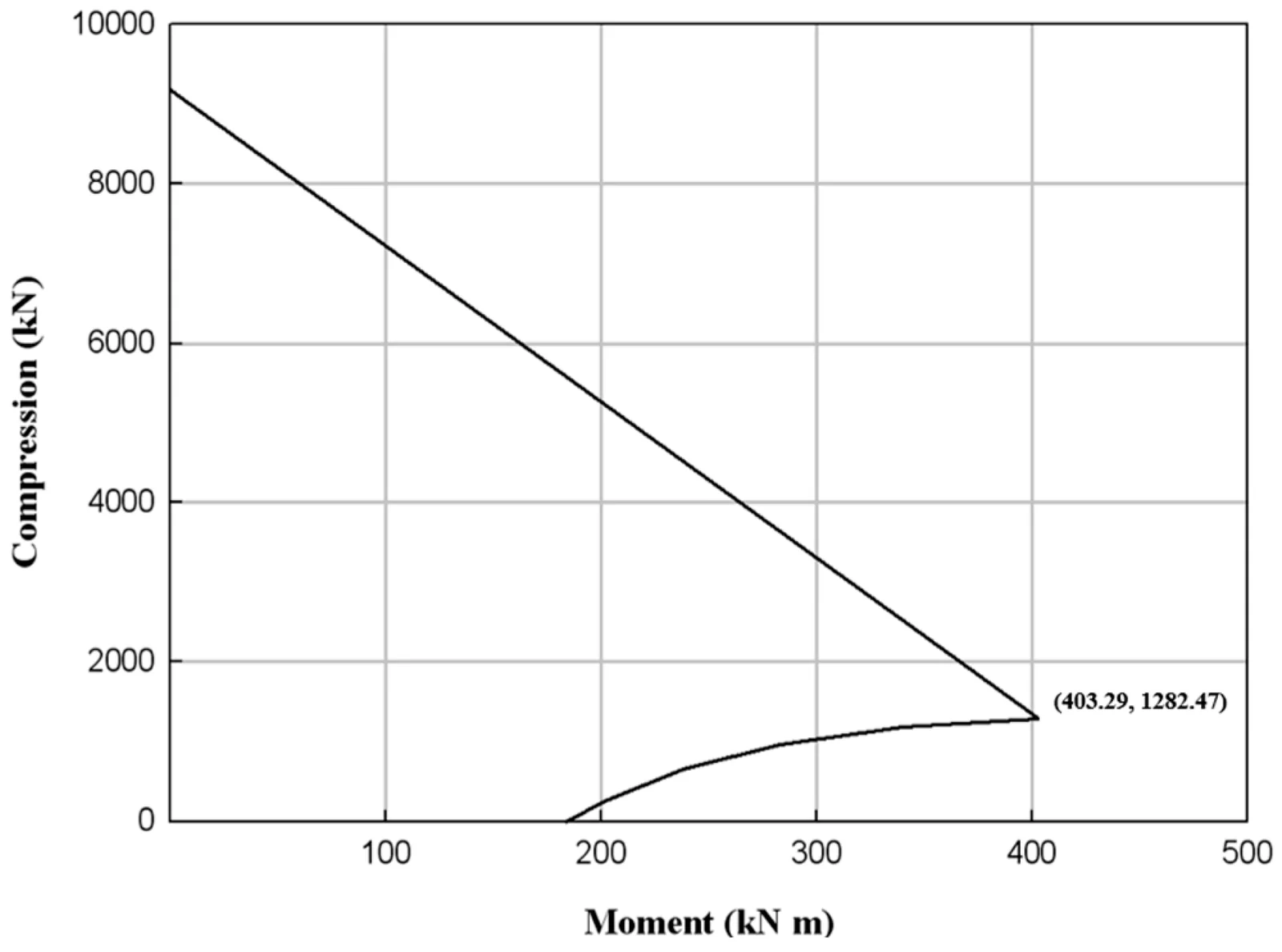
P–M interaction diagram for the 400 mm diameter HCFFT specimen.

The P–M interaction diagrams for the HCFFT specimens with diameters of 300 mm and 400 mm, fabricated and tested in this study, are presented in Figure 15 and Figure 16, respectively.

The P–M interaction is significantly influenced by the compressive strength of concrete. Therefore, the specified concrete compressive strength was selected as an independent variable, as shown in Figure 15. As illustrated in Figure 15, the flexural strength increases with increasing concrete compressive strength. Furthermore, for a given concrete compressive strength, the flexural strength increases as the thickness of either PFRP or FFRP increases. This trend can be attributed to the increased contribution of the FRP tube to the flexural resistance of the HCFFT section as its thickness increases. Moreover, increasing the thickness of the PFRP tube is more effective in enhancing the flexural strength than increasing the thickness of the FFRP tube because PFRP possesses higher mechanical strength than FFRP.

In this study, the findings reported by Chung et al. [18] were referenced to compare the structural capacities of the CFT and HCFFT. The comparison was conducted between a CFT reinforced with a steel tube having a diameter of 400 mm and a thickness of 9 mm and the HCFFT reinforced with FFRP tubes of the same diameter and thicknesses of 2.8, 4.2, and 5.4 mm. The compressive strength and flexural strength were evaluated using Equations (1) and (2), respectively.

The analytical results indicated that the compressive and flexural strengths of the HCFFT were comparable to or greater than those of the CFT. These findings demonstrate that the HCFFT can serve as a more efficient pile member than CFT when used in pile applications. A comparison of the results obtained for the HCFFT and CFT is presented in Table 8.

## 5. Conclusions

This paper presented the results of an investigation into the axial compression–flexure interaction behavior of hybrid FRP–concrete composite piles. The following conclusions were drawn:

(1) A new type of pile with a hybrid FRP cross-section (i.e., a hybrid FRP–concrete composite member) was developed through experimental and analytical investigations. The hybrid FRP–concrete composite member consists of core concrete, an outer FFRP tube, and PFRP unit modules located between the core concrete and the FFRP tube. The PFRP unit modules enhance the flexural load-carrying capacity, whereas the outer FFRP tube and the concrete core improve the axial load-carrying capacity.
(2) Compression tests were conducted on CFFTs to evaluate the axial load-carrying capacity of HCFFTs. Based on the experimental results, the relationship between the confinement ratio and the strength ratio of HCFFTs was established, and an equation for predicting the axial compressive load capacity of HCFFTs was proposed.
(3) The flexural behavior of HCFFTs was investigated using the results of a previous experimental study. Based on the experimental results, the relationship between the FFRP thickness and the flexural load-carrying capacity of HCFFTs was established. In addition, equations for predicting the flexural load-carrying capacity of HCFFTs were proposed using the plastic stress distribution method and the strain compatibility method. The analytical results obtained by two methods are compared with the experimental results.
(4) For the prediction of the flexural load-carrying capacity of the HCFFT, the plastic stress distribution method showed significantly better agreement with the experimental results than the strain compatibility method.
(5) Finally, the P–M interaction curves of HCFFTs, which are essential for structural design, are presented. In addition, a parametric study was conducted to investigate the P–M interaction behavior of HCFFTs. The results indicate that the parameters considered in this study, including the concrete compressive strength, are closely related to the axial compressive and/or flexural strengths of HCFFTs.

## Figures and Tables

**Table 1:** Concrete compressive strength and curing period.

Concrete Design Compressive Strength	Curing Period (Days)	Number of Specimens
21	7	5
	14	5
	28	5
30	7	5
	14	5
	28	5
40	7	5
	14	5
	28	5

**Table 2:** Result of concrete compressive strength test.

Concrete Compressive Strength $\left(f_{ck},\right)$ MPa	Curing Period (Days)	Specimen Number	Failure Load (kN)	Ave. Failure Load (kN)	Ave. Failure Stress/Compressive Strength (%)
21	7	21-7-1	270.83	241.57 (13.67 MPa)	65.11
		21-7-2	141.37		
		21-7-3	250.54		
		21-7-4	203.35		
		21-7-5	278.62		
	14	21-14-1	293.78	243.41 (13.77 MPa)	65.59
		21-14-2	265.79		
		21-14-3	242.20		
		21-14-4	187.07		
		21-14-5	222.23		
	28	21-28-1	318.12	338.91 (19.18 MPa)	91.33
		21-28-2	337.44		
		21-28-3	366.52		
		21-28-4	280.52		
		21-28-5	361.17		
30	7	30-7-1	516.02	521.85 (29.53 MPa)	98.43
		30-7-2	503.13		
		30-7-3	454.26		
		30-7-4	555.01		
		30-7-5	546.39		
	14	30-14-1	569.33	567.69 (32.12 MPa)	107.08
		30-14-2	581.63		
		30-14-3	587.12		
		30-14-4	337.74		
		30-14-5	552.12		
	28	30-28-1	635.04	610.21 (34.53 MPa)	115.10
		30-28-2	652.10		
		30-28-3	523.94		
		30-28-4	614.38		
		30-28-5	581.21		
40	7	40-7-1	629.61	649.95 (36.78 MPa)	91.94
		40-7-2	669.96		
		40-7-3	644.15		
		40-7-4	650.56		
		40-7-5	655.15		
	14	40-14-1	725.07	664.86 (37.62 MPa)	94.06
		40-14-2	631.17		
		40-14-3	638.33		
		40-14-4	402.68		
		40-14-5	731.22		
	28	40-28-1	822.35	777.49 (44.00 MPa)	109.99
		40-28-2	757.82		
		40-28-3	835.14		
		40-28-4	752.31		
		40-28-5	744.90		

**Table 3:** Concrete mixed proportions.

Concrete Design Compressive Strength (f_ck_, MPa)	W/B (%)	S/a (%)	Unit Weight of Mixed Materials (kg/m^3^)						
			W	B	C	F/A	S	G	A.D
21	52.0	51.0	165	317	286	31	928	909	2.22
30	40.0	48.0	168	420	378	42	827	913	3.15
40	34.0	46.0	170	500	450	50	759	908	4.25

**Table 4:** Result of CFFT compression strength test (ø150 mm × 300 mm).

FFRP Thickness ($t_{ffrp},\;mm$)	Specimen No.	Failure Load (kN)		
		$f_{ck}\;=\;21$ MPa	$f_{ck}\;=\;30$ MPa	$f_{ck}\;=\;40$ MPa
2.8	1	1257	1242	1163
	2	1269	1010	1227
	3	1282	1377	1152
	Average	1269 ± 10.20	1210 ± 151.56	1181 ± 33.06
4.2	1	1975	2090	2105
	2	1873	2255	2138
	3	1885	2060	2235
	Average	1911 ± 45.51	2135 ± 85.73	2159 ± 55.17
5.6	1	2323	2698	2492
	2	2190	2788	2920
	3	1990	2883	2860
	Average	2168 ± 136.86	2790 ± 75.53	2757 ± 189.21

**Table 5:** Comparison between experimental results and compressive strength prediction equation.

Standard Design Strength of Concrete ($f_{ck}$, MPa)	FFRP Thickness ($t_{f}$, mm)	Average Failure Strength		
		Equation (3) (MPa) (*P_HCFFT_*)	Experiment (MPa) (*P_Exp_*)	*P_HCFFT_*/*P_Exp_*
21	2.8	70.50	46.13	1.53
	4.2	87.52	57.67	1.52
	5.6	93.72	60.74	1.54
30	2.8	68.85	51.40	1.34
	4.2	93.69	69.25	1.35
	5.6	110.66	67.86	1.63
40	2.8	68.05	56.75	1.20
	4.2	94.35	67.03	1.41
	5.6	109.80	72.95	1.51

**Table 6:** Result of HCFFT finite element analysis (ø150 × 300 mm).

Standard Design Strength of Concrete ($f_{ck}$, MPa)	FFRP Thickness ($t_{f}$, mm)	Failure Strength (MPa)		
		Experiment ($f_{e}$ )	FEA ($f_{f}$)	Error (%)
21	2.8	46.13	46.07	−0.14
	4.2	57.67	56.88	−1.39
	5.6	60.74	56.48	−7.54
30	2.8	51.40	47.06	−9.22
	4.2	69.25	62.64	−10.55
	5.6	67.86	58.25	−16.49
40	2.8	56.57	49.47	−14.35
	4.2	67.03	57.74	−16.10
	5.6	72.95	59.85	−21.88

**Table 8:** Comparison of flexural test results and predicted flexural strengths for HCFFT specimens.

Load Type	CFT	HCFFT		
		2.8	4.2	5.6
Compression (kN)	8802	7986	9144	10,302
Flexure (kNm)	2445	2575	2663	2754

## Data Availability

The original contributions presented in this study are included in the article. Further inquiries can be directed to the corresponding author.
